# Spatial shopping behavior during the Corona pandemic: insights from a micro-econometric store choice model for consumer electronics and furniture retailing in Germany

**DOI:** 10.1007/s10109-023-00408-x

**Published:** 2023-04-03

**Authors:** Thomas Wieland

**Affiliations:** Institute of Geography and Geoecology, Karlsruhe Institute of Technology (KIT), Human Geography, Kaiserstr. 12, 76131 Karlsruhe, Germany

**Keywords:** Spatial shopping behavior, Multi-channel retailing, Online shopping, Store choice model, Retail location theory, Corona pandemic, C35, C51, C81, R32, L81

## Abstract

During the COVID-19 pandemic, e-commerce’s market share has increased dramatically, a phenomenon attributable to not only lockdowns but to voluntary changes in shopping behavior as well. The current study examines the main determinants driving shopping behavior in the context of both physical and online store availability, and investigates whether specific drivers have changed during the pandemic. The study aims to test whether fear of infection and mandatory wearing of face masks in shops have influenced consumer channel choice. The empirical analysis focuses on two product types (consumer electronics, furniture), with empirical data collected via a representative consumer survey in three German regions. The statistical analysis was performed using a hurdle model approach and the findings are compared to those of a study related to pre-pandemic shopping. The results show that the determinants of shopping behavior have largely not changed. Channel choice can be explained by shopping attitudes, age, and partially, by place of residence of consumers. Store choice is determined primarily by shopping transaction costs and store features. Fear of infection and the mandatory wearing of face masks exhibit minimal influence on channel choice, if any. The importance of cross-channel integration of stores/chains has decreased significantly, while average travel times for in-store purchases have declined.

**Supplementary Information:**

The online version contains supplementary material available at 10.1007/s10109-023-00408-x.

## Introduction

Online retailing has experienced a huge boost in the context of the COVID-19 pandemic. Of course, the lockdowns enforced in many European countries immediately impacted shopping behavior due to the closure of “non-essential” retail stores. However, these lockdowns were temporary and cannot be considered solely responsible for the enormous increase in the relevance of online retailing. Several studies suggest that during the pandemic—independent of any lockdowns—voluntary behavioral changes such as reductions in shopping mobility and switching to e-shopping due to fear of coronavirus infection occurred (e.g., Chenarides et al. 2021; Goolsbee and Syverson 2021; Jacobsen and Jacobsen 2020; Jiao and Azimian 2021; Shamshiripour et al. 2021). In addition, in many European countries, the face mask mandate in retail stores, which many consumers found uncomfortable or categorically rejected, may have impacted shopping behavior (Bonial 2020; Knotek et al. 2020; Taylor and Asmundson 2021). In view of the above findings, one can assume that the pandemic situation has also influenced spatial shopping behavior, independently of forced business closures, particularly in favor of e-commerce. Research on shopping behavior has so far focused on either store choice in physical retailing or channel choice in multi-channel retailing, using either store choice or channel choice models (see Sect. 2). The studies mentioned above, on the other hand, refer to changes in (shopping) behavior in the COVID-19 pandemic, but without modeling spatial shopping behavior. Unfortunately, there have not yet been any model-based studies on spatial shopping behavior in the context of the COVID-19 pandemic. This study aims to fill this gap.

Based on an empirical, model-based store choice approach, the current study attempts to answer the following research questions: (1) What are the main drivers of spatial shopping behavior given the availability of both physical *and* online stores? (2) Have the determinants of spatial shopping behavior changed during the pandemic? (3) Have fear of infection and the obligation to wear a face mask in retail stores influenced channel choice in favor of online retailing? In order to answer these questions, Wieland’s (2021a, b, 2023) study design on micro-econometric modeling of spatial shopping behavior in a multi-channel context is here repeated for the purposes of comparison. The empirical analysis focuses on consumer electronics retailing and furniture retailing. Empirical data on shopping behavior was collected via a representative consumer survey conducted in three German regions. The econometric modeling approach follows on from Wieland’s (2021a, 2023) previous studies on consumer electronics and furniture shopping; it is based on the hurdle model, which is used frequently to answer questions about individual consumption decisions and with which it is possible to split human behavior into an “if” and a “how” decision. Since the model is designed for micro data, it is possible to include individual consumer characteristics of both an objective and subjective nature. The current model considers shopping transaction costs, store characteristics, and psychographic consumer characteristics (shopping attitudes). In order to investigate the influence of the pandemic on shopping decisions, attitudes including fear of infection and the perceived burden of masks were also surveyed and integrated into the model as explanatory variables.

The study approach demonstrates that online *and* physical retailers can be incorporated into a store choice model for spatial shopping behavior, which is originally derived from retail location theory and developed for physical retail locations alone. The fact that such a model can also be used for the multi-channel context represents a theoretical and methodological extension of the analysis concepts in retail geography. With respect to the comparison of shopping behavior before and during the pandemic, it can be shown that the determinants of shopping behavior have largely not changed. While channel choice is mainly explained by the age of the consumers, their attitudes and, in some cases, their place of residence, transaction costs and store features have a major influence on store choice. The pandemic situation—in the sense of fear of infection and mask aversion—has only a minimal impact on shopping behavior, if at all.

The paper is structured as follows. Section 2 describes the literature on spatial shopping behavior in the context of retail location theory, multi-channel shopping behavior, and voluntary behavior changes during the pandemic. Section 3 outlines the modeling approach, the explanatory variables, and their expected impact, as well as the data collection procedure. In Sect. 4, the empirical findings are presented in terms of descriptive results, intermediate results toward the psychographic consumer attributes, and model results for the three survey areas. Section 5 summarizes the main conclusions of this study and discusses some limitations of the study.

## Literature review

### Spatial shopping behavior in retail location theory

According to Brown (1993), there are four domains of retail location theory: (1) central place theory (and its extensions), (2) spatial interaction models, (3) theories and models of retail agglomeration, and (4) bid rent theory—whereby the first three focus on spatial shopping behavior. *Central place theory* (CPT) (Christaller 1933) and its successors focus spatial shopping behavior by emphasizing the respective roles of accessibility and (consumer) transport costs on the choice of a shopping location. According to CPT, consumer demand for a given good decreases with increase in transport costs (distance-dependent demand), and consumer sensitivity toward transport costs reduces with decrease in purchasing frequency of the desired good. CPT was first adopted worldwide in the 1960s and has been empirically tested and theoretically expanded over a period of several decades. Extensions of this theory aim to make it more dynamic (e.g., Eaton and Lipsey 1982; Ghosh 1986; Lange 1973), with some emphasizing the importance of multipurpose shopping in particular: An important argument emerging from these extensions is that retail locations with greater potential for multipurpose shopping are preferred by consumers, even if visiting them incurs higher transport costs, a factor which may be regarded as the explicit implementation of agglomeration effects (here: urbanization economies) within CPT (Eaton and Lipsey 1982; Ghosh 1986; Lange 1973). The CPT received new attention and further expansions of its theoretical ideas through the emergence of the so-called “*New Economic Geography*” (NEG). The basic assumptions regarding spatial consumer behavior were either retained or expanded by aspects such as multipurpose and/or comparison shopping (e.g., Fujita and Thisse 2002).

Independent from CPT, *spatial interaction models* (SIM) for retailing incorporating similar theoretical assumptions toward spatial consumer behavior (e.g., with respect to accessibility) have been developed (e.g., Reilly 1931; Huff 1962). Originating from deterministic models for two supply locations (e.g., Reilly 1931), Huff (1962) created a probabilistic store choice model based on microeconomic assumptions. This model is formalized mathematically and estimates the probability of a (shopping) decision based on the assumptions of utility maximization and imperfect information of consumers. Consumer utility of a shopping location is assumed to be impacted by (1) travel time and (2) the location’s respective assortment and store size. Travel time has an overproportionate negative influence on store choice due to the opportunity costs involved in traveling to shopping locations. Conversely, store size increases consumer utility of visiting a store because consumers decide for a shopping location based on imperfect information, whereby, the larger the store’s assortment, the more likely it is that a consumer will obtain the desired goods. However, as consumer search and decision costs increase with increase in assortment, a larger assortment is assumed to be affected by diminishing marginal utility. In the *Huff model*, the probability that a consumer chooses a shopping location is equal to the store’s utility relative to the sum of the utilities of all shopping locations.

The Huff model has been further developed in many ways, both theoretically and in terms of application (methodology). These include, among other things, the transformation into an econometric model with which empirical shopping behavior can be analyzed, namely the *Multiplicative Competitive Interaction* (MCI) model (Nakanishi and Cooper 1974). An important extension, for example, is the *Competing Destinations Model* by Fotheringham (1985), in which an additional variable that depicts the clustering of stores operationalizes the influence of (positive) agglomeration effects or competitive effects. Further developments concern, for example, the integration of (subjective) variables regarding the shops/chains concerned (e.g., Stanley and Sewall 1976) or model alternatives for depicting multipurpose trips (e.g., O'Kelly 1981). The Huff model also plays an important role in the applied context, especially in location planning of retail companies (Berman and Evans 2013) and in spatial planning regarding *retail impact assessments* (Khawaldah et al. 2012; Müller-Hagedorn 2020). Note that the Huff model is just one of many spatial interaction models, many of which are also used to model spatial shopping behavior (e.g., Nakaya et al. 2007).

The third theoretical strain mentioned above emphasizes the role of positive agglomeration effects in retailing due to the opportunity for multipurpose and comparison shopping. Hotelling’s microeconomic model (“*principle of minimum differentiation*”) is usually considered to be the first theory of this kind (although it fails to consider multipurpose or comparison shopping). Hotelling’s model describes a duopoly in a linear market, whereby suppliers relocate to maximize their profits. The best location for both suppliers is a cluster in the middle of the market, where each supplier serves one half of the market (Hotelling 1929). While all the aforementioned theories and models represent a deductive approach, Nelson (1958) has worked solely from an inductive perspective. Based on empirical observations on shopping behavior, Nelson formulated two theories on agglomeration effects in retailing. The “*theory of cumulative attraction”* relates to competing retailers selling different product variants (e.g., shoe stores). If such stores cluster together, this enables comparison shopping, and thus, generates more customer traffic when compared to the sum of all these retailers when located in solitary locations. However, stores from different sectors may increase their joint demand if they build a cluster provided that such stores are compatible with respect to multipurpose shopping (“*rule of retail compatibility”*). Clustering of (complementary or competitive) retail establishments has been incorporated into later monopolistic competition models and subsequent NEG models (e.g., Fujita and Thisse 2002).

The SIM and its successors can also be used directly empirically, provided real data on spatial shopping behavior is available. The assumptions of CPT and SIM with respect to assortment and accessibility were often examined empirically, using either econometric market area models such as the MCI model or discrete choice models for individual decisions. Here, the assumed impacts of, inter alia, assortment and travel time have been frequently confirmed (e.g., Baviera-Puig et al. 2016; Briesch et al. 2009; Orpana and Lampinen 2003; Popkowski Leszczyc et al. 2004; Suárez-Vega et al. 2015; Tihi and Oruc 2012; Wieland 2015, 2018). Positive agglomeration effects have only been examined in a few store choice studies with the related findings failing to demonstrate congruency and rather depending on the examined product types (Orpana and Lampinen 2003; Tihi and Oruc 2012; Wieland 2015). Importantly, this family of retail location theory and the associated empirical work is geared solely toward physical retailing.

### Multi-channel shopping behavior

Research into multi-channel shopping behavior typically deals with consumer shopping channel choice (online vs. in-store) or online shopping frequency. A large branch of the literature dealing with multi-channel shopping behavior focuses on channel-specific shopping *transaction costs*. These costs include aspects such as travel time to physical stores, delivery charges and delivery time (waiting time) in online retailing, or search and information costs in both channels (Chintagunta et al. 2012). Several studies have shown empirically that decreasing travel time to physical shopping locations and increasing delivery charges and delivery time decrease the likelihood of online shopping (Chintagunta et al. 2012; Hsiao 2009; Marcucci et al. 2021; Marino et al. 2018; Schmid and Axhausen 2019). However, the informative value of such studies is limited since they are based either on survey experiments (Hsiao 2009; Marcucci et al. 2021; Schmid and Axhausen 2019) or on empirical data from individual companies (Chintagunta et al. 2012; Marino et al. 2018).

One explicit geographic perspective on online shopping deals with spatial differences in shopping channel choice based on two competing hypotheses. The *innovation-diffusion hypothesis* assumes that urban residents are more likely to buy online due to a greater openness to new technologies. In contrast, the *efficiency hypothesis* states that consumers in rural areas tend to buy more online because of the (assumed) lower accessibility to physical shopping locations (Cao et al. 2013). The first hypothesis has typically been confirmed in previous studies (Cao et al. 2013; Farag et al. 2006; Zhen et al. 2018); however, there are several more recent studies which no longer find a higher online affinity in (large) cities (Clarke et al. 2015; Beckers et al. 2018). In contrast, the efficiency hypothesis was frequently confirmed. Several studies have found that spatial proximity to competing stores decreases the likelihood of online shopping (Cao et al. 2013; Clarke et al. 2015; Dijst et al. 2008; Farag et al. 2006; Zhai et al. 2017; Zhen et al. 2018). However, these effects have been found to differ between region types (Cao et al. 2013) and product types (Farag et al. 2006; Zhen et al. 2018). Both the transaction cost perspective and the efficiency hypothesis relate to accessibility of physical stores. As accessibility and opportunity costs of traveling are important aspects in retail location theory, there is an obvious connecting point between store choice and channel choice studies. In principle, delivery fees and waiting time which occur in e-shopping might be regarded as the equivalent of the opportunity costs of in-store shopping trips.

Several studies investigating channel choice have also included psychographic consumer attributes, in particular *attitudes*, which are subjective perceptions of reality (here: shopping channels), regardless of whether these perceptions are correct or not (Dijst et al. 2008). Typically, latent variables are inferred from individual statement items which relate, for example, to the perceived risks of online purchases (e.g., with respect to credit card fraud or privacy) or to the pleasure of shopping in physical stores. The inferred variables then represent higher-level individual attitudes with respect to the shopping channels. Several studies have shown that a positive attitude toward e-commerce (e.g., low risk aversion) increases the likelihood of shopping online, whereas enjoyment of in-store shopping reduces online affinity (Dijst et al. 2008; Schmid and Axhausen 2019; Zhai et al. 2017).

Additionally, most studies include socio-demographic consumer attributes. One obvious tendency is that consumers of a younger age tend to buy more online than older consumers, which may be attributed to a younger consumer’s experience with information and communication technology (ICT). Furthermore, the likelihood of buying online was found to be higher for consumers with higher education and/or income, male consumers, and consumers in employment (Beckers et al. 2018; Clarke et al. 2015; Farag et al. 2006).

Recently, a few studies that integrate online retailing into a store choice framework have emerged. The model by Beckers et al. (2021) approaches behavior on an aggregated level (similar to the Huff model), and thus, fails to allow assessment of the impact of individual consumer characteristics. Suel and Polak (2017) investigated channel, store, and travel mode choice with respect to grocery retailing simultaneously. They found few significant socio-demographic effects on channel choice but did demonstrate a higher likelihood for higher social classes to visit expensive stores and shop online. With respect to store choice, store size increases and travel time (driving, public transport, or walking) reduces the likelihood of a store being chosen, which is in line with previous store choice studies. Using a store choice and expenditure model, Wieland (2021a, b, 2023) has combined the aforementioned approaches of channel and store choice. Results have shown that channel choice is mainly influenced by shopping attitudes, place of residence, and age of the consumers. Consumers with a “pro online” attitude, urban residents, and younger consumers were found to exhibit a higher likelihood for e-shopping. Store choice and expenditures were primarily explained by store features such as assortment size, cross-channel integration, and transaction costs (travel time, delivery charges, and delivery time).

### Shopping behavior changes in the COVID-19 pandemic

The dramatic increase in market share for online retailers during the COVID-19 pandemic has occurred due to changes in individual shopping behavior. Of course, these changes may be a direct result of government-imposed restrictions, in particular, lockdowns including stay-at-home orders and closing of “non-essential” businesses. When physical retail stores are closed during a lockdown (as imposed in most Western countries), there is no opportunity for shopping there at all. However, changes in shopping behavior may also occur on a *voluntary* basis in the absence of lockdowns and may result from individual fear of infection (Goolsbee and Syverson 2021). From a psychological perspective, fear of infection may motivate people to change their behavior in terms of avoiding situations with risk of infection and in establishing preventive actions (Stangier et al. 2022).

For example, with respect to Germany, Stangier et al. (2022) investigated the impact of fear of infection and knowledge about the virus on (reported) behavioral changes. For the measurement of fear of infection, they utilized the *Perceived Vulnerability to Disease* (*PVD*) scale consisting of two latent variables, “perceived infectability” and “germ aversion,” both of which were inferred from a survey (Duncan et al. 2009). The results demonstrated that both perceived infectability and germ aversion significantly increased preventive behavior. In addition, germ aversion significantly reduced risk behavior, which corresponded to reducing physical contacts. However, the Stangier et al. study did not explicitly investigate shopping trips and therefore could not assess whether behavioral changes were actually implemented.

There are various studies on spatial mobility that show a significant decline in physical shopping trips in the context of the pandemic. With respect to the first wave of infections in spring 2020, Jacobsen and Jacobsen (2020) analyzed Google mobility data from the USA and found a substantial reduction in the second half of March 2020 with respect to visits to transit stations, retail and recreation facilities, grocery stores, and pharmacies. These changes were found to be of a similar magnitude in states with and without a formal lockdown. This reduction of visits to grocery stores and pharmacies—both of which were never closed—was approximately 16% and 15%, respectively. Goolsbee and Syverson (2021) investigated spatial consumer behavior in the USA based on mobile phone data from March to May 2020, including US counties with and without formal lockdowns. Their results showed a large decline in over-all mobility, of about 60%, with the estimated impact of formal lockdowns being found to be approximately 7% only. This decline impacted, inter alia, clothing, and do-it-yourself stores as well as grocery stores and pharmacies. The Jiao and Azimian (2021) study covered the second phase of the pandemic and employed a survey on mobility behavior, which was conducted in the absence of formal lockdowns in the USA (October 2020). This study found a significant reduction of trips to retail stores and for trips by public transport, with stronger effects in the group of people aged 35 or older.

In their survey from April to June 2020 in Chicago, Shamshiripour et al. (2021) showed that people’s tendency to buy groceries online grew substantially during the first wave of the pandemic, regardless of the formal lockdown. For several other major US cities, Chenarides et al. (2021) found the same trend to be occurring, with respondents’ main reasons for not shopping in-store being “scared of COVID-19” and “feeling unsafe.” During this same time frame, Rossetti et al. (2022) also conducted a stated choice experiment investigating grocery store choice under pandemic conditions. They examined the effects of infection control measures while shopping, and found, among other things, that consumers accept certain inconveniences (e.g., longer waiting time, queues in front of the store) if, in return, infection control measures (e.g., face masks, limitation of the number of customers) are in place. All in all, these studies suggest that, at least in the early phase of the pandemic, there were voluntary changes in behavior to reduce risk and that consumers’ online affinity subsequently increased as a result of fear of infection.

In addition, the face mask mandate in retail shops, which applied in many European countries from April 2020 to (mostly) April 2022, may have had an impact on shopping behavior. As studies have shown, a certain part of the population rejects face masks for ideological reasons or at least finds them uncomfortable (Knotek et al. 2020; Matusiak et al. 2020; Taylor and Asmundson 2021). It is therefore plausible to assume that people who are negatively affected by wearing a mask avoid situations in which a mask is mandatory. However, it has not yet been empirically investigated whether consumers are switching or have switched to e-shopping because of this. There are only indications that consumers are avoiding retail locations where masks are mandatory. In a German survey from October 2020 conducted by Bonial (2020), 40% of the respondents stated that they shop less frequently in city centers because of the mask mandate in retail stores. In any case, the mandate might have encouraged, at least some, consumers to buy online rather than in-store.

## Modeling approach and data collection

### Modeling approach

The econometric strategy in this study is based on the modeling approach outlined in Wieland (2021a, b, 2023), which incorporates an adaptation of the *hurdle model* (Mullahy 1986) applied to (spatial) shopping behavior. The hurdle model is a special kind of count data model which was designed for excess of zeros in the dependent variable. This is a two-part model. In the first part, a binary probability model determines whether there is a null or a nonzero outcome; the positive outcomes are treated in the second part of the model using a (truncated) count data model. Thus, although the model is designed on the basis of mathematical principles (heavily skewed dependent variables), it nevertheless implicitly represents a two-step decision process (“if”- and “how”-decision), which is why the model is often used to analyze individual demand in micro-econometric studies (Greene 2012; Cameron and Triverdi 2005). Similar to previous store choice and channel choice models, the underlying rationale for the current model is probabilistic choice behavior which assumes consumer utility maximization. The first model part (usually referred to as “*participation equation*”) deals with whether consumer choice for *something* (here: shopping at a given store) is performed and measures the probability of that event occurring. Mathematically, this is the probability that the dependent variable (here: a consumer’s sum of expenditure at a given store) is greater than zero. The second part of the model (usually referred to as “*intensity equation*” or sometimes “*expenditure equation*”) is employed only for observations greater than zero and deals with the issue of *how* something is performed (here: a consumer’s sum of expenditure at a given store which was chosen before) (Cameron and Triverdi 2005; Wieland 2021a, b, 2023).

The representation provided here follows that of Wieland (2021a, b, 2023). The dependent variable in the model equals the expenditures of consumer *I* at (physical or online) store *j*, denoted *S*_*ij*_ hereafter. This variable represents both store and consumer attributes. The utility of store *j* (*j* = 1,…,*J*) for consumer *i* (*i* = 1,…,*I*) consists of an explained part (representative utility), *V*_*ij*_, and an unobserved part, the error term, *ε*_*ij*_:

1 $${U}_{ij}={V}_{ij}+{\varepsilon }_{ij}$$

The representative utility of store *j* for consumer *i*, *V*_*ij*_, is a combination of the utility of the channel and the utility of the individual store, with the first part reflecting the consumer's affinity for e-shopping and the second part comprising the most important attributes of the store. To emphasize this distinction, we will henceforth denote the total utility ${V}_{ij}^{P}$ , the store utility ${V}_{ij}^{S}$, and the channel utility ${V}_{ij}^{C}$:

2 $${V}_{ij}^{P}={V}_{ij}^{S}+{V}_{ij}^{C}$$

The part ${V}_{ij}^{C}$  contains variables which are assumed to have a significant impact on channel choice, which includes socio-demographic, spatial, and attitudinal characteristics of consumers (see Sect. 2.2). The impact of a variable on channel choice is assessed using interaction terms incorporating the variable describing a consumer characteristic and a dummy variable (*DO*_*j*_) indicating whether store *j* is an online store (*DO*_*j*_ = 1) or not (*DO*_*j*_ = 0). Table 1, section “Independent variables A,” shows the independent variables considered relevant to channel choice. These are the variables used in the previous studies (Wieland 2021a, b, 2023) but supplemented by psychographic consumer characteristics related to the SARS-CoV-2 pandemic. The utility of the online channel for the individual consumer *i*, ${V}_{ij}^{C}$, is thus defined as:

3 $${V}_{ij}^{C}={\beta }_{1}D{O}_{j}+{\beta }_{2}D{25}_{i}+{\beta }_{3}D{65}_{i}+{\beta }_{4}D{m}_{i}+{\beta }_{5}D{E}_{i}+{\beta }_{6}D{L}_{i}+{\beta }_{7}P{O}_{i}+{\beta }_{8}F{I}_{i}+{\beta }_{9}G{A}_{i}+{\beta }_{10}M{A}_{i}+{\beta }_{11}\left(D{O}_{j}*D{25}_{i}\right)+{\beta }_{12}\left(D{O}_{j}*D{65}_{i}\right)+{\beta }_{13}\left(D{O}_{j}*D{m}_{i}\right)+{\beta }_{14}\left(D{O}_{j}*D{E}_{i}\right)+{\beta }_{15}\left(D{O}_{j}*D{L}_{i}\right)+{\beta }_{16}\left(D{O}_{j}*P{O}_{i}\right)+{\beta }_{17}\left(D{O}_{j}*F{I}_{i}\right)+{\beta }_{18}\left(D{O}_{j}*G{A}_{i}\right)+{\beta }_{19}\left(D{O}_{j}*M{A}_{i}\right)$$

where *DO*_*j*_ denotes whether store *j* is an online store (dummy variable), *D25*_*i*_ and *D65*_*i*_ indicate whether consumer *i* is under 25 years old or at least 65 years old (dummy variables), *Dm*_*i*_ indicates whether consumer *i* is male (dummy variable), *DE*_*i*_ indicates whether consumer *i* is employed (dummy variable), *DL*_*i*_ indicates whether consumer *i* lives in a large city (dummy variable), *PO*_*i*_ is the value of the latent attitude variable (“pro online” attitude) of consumer *i*, *FI*_*i*_ and *GA*_*i*_ are the values of consumer *i*'s latent variables related to fear of infection (“perceived infectability” and “germ aversion”), *MA*_*i*_ is the score for consumer *i*'s rejection of face masks when shopping, *β*_*1*_, …, *β*_*19*_ are the regression coefficients to be estimated.

**Table 1:** Variables in the models

Variable	Notation	Description
*Independent variables A (Channel choice)*		
Online store	$D{O}_{j}$	Dummy variable indicating that store *j* is an online store [1] or a physical store [0]
Large city	$D{L}_{i}$	Dummy variable indicating if consumer *i* lives in a large city [1] or not [0]
LV (shopping attitude: pro online)	$P{O}_{i}$	Latent variable (factor) extracted from 15 attitude items (4-point Likert scale)
Age < 25	$D{25}_{i}$	Dummy variable indicating if consumer *i* is under 25 years old [1] or not [0]
Age > 65	$D{65}_{i}$	Dummy variable indicating if consumer *i* is at least 65 years old [1] or not [0]
Gender	$D{m}_{i}$	Dummy variable indicating if consumer *i* is male [1] or not [0]
Employed	$D{E}_{i}$	Dummy variable indicating if consumer *i* is employed [1] or not [0]
Perceived infectability	$F{I}_{i}$	Latent variable (factor) 1 extracted from 13 attitude items (Perceived vulnerability to disease scale; 4-point Likert scale)
Germ aversion	$G{A}_{i}$	Latent variable (factor) 2 extracted from 13 attitude items (Perceived vulnerability to disease scale; 4-point Likert scale)
Face mask inconvenience	$M{A}_{i}$	Perceived inconvenience of face masks in retail shops (4-point Likert scale)
*Independent variables B (Store choice)*		
Assortment	${A}_{j}=\sum_{c=1}^{C}item{s}_{{c}_{j}}$	Number of items of store *j* over all *C* categories [quantity]
Travel time	${t}_{ij}$	Car travel time from consumer *i* to physical store *j* [minutes]; *t*_*ij*_ = 0 for online stores
Delivery time *	${st}_{j}$	Delivery time of online store *j* [days]; *st*_*j*_ = 0 for physical stores
Delivery charges	$s{c}_{j}$	Delivery charges of online store *j* [EUR]; *sc*_*j*_ = 0 for physical stores
Delivery charges based on order value ^+^	$Dsc{o}_{j}$	Dummy variable indicating whether delivery charges of store *j* (or the chain *j* belongs to) changes with order value [1] or not [0]
Free delivery from a certain order value^+^	$Dsc{f}_{j}$	Dummy variable indicating whether delivery charges of store *j* (or the chain *j* belongs to) are free from a certain order value [1] or not [0]
Integrated online shop *	$D{I}_{j}$	Dummy variable indicating whether the store *j* (or the chain *j* belongs to) is engaged in multi-channeling with an integrated online shop [1] or not [0]
BIPOS*	$D{C}_{j}$	Dummy variable indicating whether the store *j* (or the chain *j* belongs to) offers BIPOS [1] or not [0]
Cross-channel retailer^+^	$D{CC}_{j}$	Dummy variable indicating whether the store *j* (or the chain *j* belongs to) is a cross-channel retailer [1] or not [0]
Located in shopping mall*	$DS{M}_{j}$	Dummy variable indicating if the physical CE store *j* is located in a shopping mall [1] or not [0]
Clustering with competitors	$${C}_{j}=\sum_{\begin{subarray}{c}k=1\\ k\ne j\end{subarray}}^{K}{A}_{k}{d}_{jk}^{-\varphi }$$	Hansen accessibility for spatial proximity of store *j* to all other *K* competitors (airline distance *d*_*jk*_ with weighting exponent *φ* = 2); *C*_*j*_ = 0 for online stores
Consumer electronics chains*	$DSa{t}_{j}$, $DMe{d}_{j}$, $DEx{p}_{j}$, $DEu{r}_{j}$,$DE{P}_{j}$	Dummy variables indicating if the physical/online CE store belongs to the corresponding chain/company [1] or not [0] (*Saturn*, *Media Markt*, *Expert*, *Euronics*, *EP*)
Furniture chains^+^	$DJYS{K}_{j}$, $DIKE{A}_{j}$, $DPoc{o}_{j}$, $DBos{s}_{j}$, $DMo{m}_{j}$, $DSc{o}_{j}$, $DXX{L}_{j}$, $DAma{z}_{j}$, $Deba{y}_{j}$	Dummy variables indicating if the physical/online furniture store belongs to the corresponding chain/company [1] or not [0] (*JYSK*, *IKEA*, *Poco*, *SB Möbel Boss*, *Mömax*, *Sconto*, *XXXLutz*, *Amazon*, *eBay*)
Not full range provider*	$Dnf{r}_{j}$	Dummy variable indicating if the physical/online CE store is a full range provider [0] or not [1]
Expenditures	${S}_{i}=\sum_{j=1}^{n}{S}_{ij}$	Sum of all obtained expenditures of consumer *i*

Variables marked with * only enter the store utility equation for CE stores, see Eq. (4). Variables marked with ^+^ enter the equation for furniture stores only, see Eq. (5)

The coefficients of interest are those related to the interaction terms which include place of residence, the “pro online” attitude, and the pandemic-related variables. According to the innovation-diffusion hypothesis, urban residents exhibit a higher likelihood of buying online. The dummy variable *DL*_*i*_ indicates whether consumer *i* lives in a large city, whereby “large city” is understood to mean a city with at least 100,000 inhabitants, which was taken from the official spatial classification in Germany (BBSR 2022). Thus, the coefficient *β*_*15*_ for the interaction term *DO*_j_*DL_*i*_ is expected to be significant and positive. The variable *PO*_*i*_ represents the “pro online” attitude of consumer *i*, as used in Schmid and Axhausen (2019) and Wieland (2021a, b, 2023) and is inferred from 15 statements provided by surveyed consumers (see Sect. 3.2). A “pro online” attitude is expected to increase the likelihood of buying online, and thus, coefficient *β*_*16*_ of the interaction term *DO*_j_*PO_*i*_ is expected to be significant and positive.

To account for possible impacts of the pandemic on channel choice, three variables have been introduced into the channel choice part of the utility equation, the first two of which reflect individual fear of infection of consumer *i* (*FI*_*i*_: “perceived infectability,” *GA*_*i*_: “germ aversion”) and the third the rejection of face masks when shopping (*MA*_*i*_). The first two are inferred from the *Perceived vulnerability to disease* scale, as used by Stangier et al. (2022), and the last is simply a statement reflecting the perceived inconvenience of face masks in retail shops (see Sect. 3.2). If fear of infection, as defined here, impacts shopping behavior such that physical stores are avoided and online buying is preferred with the aim of reducing infection risk, the two coefficients *β*_*17*_ and *β*_*18*_, which relate to the interaction terms *DO*_j_*FI_*i*_ and *DO*_j_*GA_*i*_, should be significant and positive (i.e., the higher the fear of infection, the more likely an online purchase). Similarly, if the face mask mandate in retail shops encouraged consumers to buy online instead of in-store, coefficient *β*_*19*_ of the interaction term *DO*_j_*MA_*i*_ should be significant and positive.

In order to ensure that socio-demographic characteristics of consumers are not decisive, the recorded characteristics (age, gender, employment status) were included in the equation as control variables: The dummy variables *D25*_*i*_ and *D65*_*i*_ show whether the interviewed consumer *i* is under 25 years old or at least 65 years old. The variable *Dm*_*i*_ indicates the gender of the consumer (1 = male, 0 = female), and *DE*_*i*_ indicates whether consumer *i* is employed. For example, if *β*_*12*_ of the interaction term *DO*_j_*D65_*i*_ is significant and negative, it means that people aged 65 and over buy significantly less online.

Store utility (${V}_{ij}^{S}$) includes a set of explanatory variables stemming from both store choice and multi-channel shopping behavior studies and describes the shopping alternatives and the corresponding shopping transaction costs (see Sections 2.1, 2.2). Table 1, section “Independent variables B,” lists the independent variables considered relevant to store choice. These are the variables used in previous studies (Wieland 2021a, b, 2023). Please note that the store utility equations for consumer electronics and furniture are not completely identical because of some industry-specific circumstances that need to be considered. For CE stores, the utility of store *j* for the individual consumer *i*, ${V}_{ij}^{S}$, is defined as (Wieland 2021a):

4 $${V}_{ij}^{S}={\gamma }_{0}+{\gamma }_{1}\mathrm{ln}{A}_{j}+{\gamma }_{2}{t}_{ij}+{\gamma }_{3}s{t}_{j}+{\gamma }_{4}s{c}_{j}+{\gamma }_{5}D{I}_{j}+{\gamma }_{6}D{C}_{j}+{\gamma }_{7}DS{M}_{j}+{\gamma }_{8} ln {C}_{j}+{\gamma }_{9}Dnf{r}_{j}+{\gamma }_{10} \left(\mathrm{ln}{A}_{j}*D{O}_{j}\right)+\sum_{g}^{G}{\delta }_{g}{D}_{{g}_{j}}\left[+\zeta \mathrm{ln}{S}_{i}\right]$$

where *A*_*j*_ denotes the number of articles of store *j*, *tij* is the travel time between consumer *i* and store *j*, *st*_*j*_ and *sc*_*j*_ denote the delivery time and the delivery costs of store *j*, *DI*_*j*_ and *DC*_*j*_ indicate whether store *j* offers an integrated online shop or the BIPOS option (dummy variables), *DSM*_*j*_ indicates whether store *j* is located in a shopping mall (dummy variable), *C*_*j*_ reflects store *j*'s spatial proximity to its competitors, *Dnfr*_*j*_ indicates whether store *j* is a non-full range provider (dummy variable), *DO*_*j*_ denotes whether store *j* is an online store (dummy variable), *D*_*gj*_ indicates whether store *j* belongs to chain *g*, *S*_*i*_ equals the all-over expenditures of consumer *i*, γ_0_, …, γ_10_, δ_g_, and ζ are regression coefficients to be estimated.

For furniture stores, the utility of store *j* for the individual consumer *i*, ${V}_{ij}^{S}$, equals (Wieland 2023):

5 $${V}_{ij}^{S}={\gamma }_{0}+{\gamma }_{1}\mathrm{ln}{A}_{j}+{\gamma }_{2}{t}_{ij}+{\gamma }_{3}s{c}_{j}+{\gamma }_{4}Dsc{o}_{j}+{\gamma }_{5}Dsc{f}_{j}+ {\gamma }_{6}DC{C}_{j}+{\gamma }_{7} ln {C}_{j}+{\gamma }_{8} \left(\mathrm{ln}{A}_{j}*D{O}_{j}\right)+\sum_{g}^{G}{\delta }_{g}{D}_{{g}_{j}}\left[+\zeta \mathrm{ln}{S}_{i}\right]$$

where *A*_*j*_ denotes the number of articles of store *j*, *t*_*ij*_ is the travel time between consumer *i* and store *j*, *sc*_*j*_ denotes the delivery costs of store *j*, *Dsco*_*j*_ and *Dscf*_*j*_ indicate whether the delivery costs of store *j* depend on the order value or whether there is free delivery above a certain order value (dummy variables), *DCCj* indicates whether store *j* has an integrated online shop *and* offers BIPOS (dummy variable), *C*_*j*_ reflects store *j*'s spatial proximity to its competitors, *DO*_*j*_ denotes whether store *j* is an online store (dummy variable), *D*_*gj*_ indicates whether store *j* belongs to chain *g*, *S*_*i*_ equals the all-over expenditures of consumer *i*, γ_0_, …, γ_8_, δ_g_, and ζ are regression coefficients to be estimated.

According to the Huff model and several empirical store choice studies, assortment size of store *j* (*A*_*j*_) is expected to have a positive but sublinear effect due to diminishing marginal utility of assortment. Thus, a significant coefficient *γ*_*1*_ between zero and one is expected. Because online stores regularly offer a considerably larger assortment than physical stores, it is tested whether there is a difference of the assortment impact between physical and online stores, assuming that this impact is lower for online stores, which means that coefficient of the interaction term *ln A*_j_*DO_*j*_ (*γ*_*10*_ in the consumer electronics model and *γ*_*8*_ in the furniture model) should be negative.

Three types of shopping transaction costs are included in the store utility function, namely travel time (*t*_*ij*_; for physical stores), delivery time (*st*_*j*_), and delivery costs (*sc*_*j*_; for online stores). According to the Huff model and other work from retail location theory, distance-dependent demand is assumed, and thus, the impact of travel time is expected to be significant and negative (coefficient of *t*_*ij*_: *γ*_*2*_ < 0). This assumption is equivalent to the findings in channel choice studies, where a better accessibility of competing physical stores decreases the likelihood of shopping online. Similarly, it is expected that delivery costs and delivery time will reduce the choice probability of a store, which would mean that coefficients *γ*_*3*_ and *γ*_*4*_ of variables *st*_*j*_ and *sc*_*j*_ in the consumer electronics model will be negative. However, there is a difference between consumer electronics and furniture stores: In the case of furniture online stores, the delivery time cannot be determined through research (because the delivery times between the individual products sometimes vary by several weeks or even months), which is why this variable (*st*_*j*_) cannot be included in the store utility equation for furniture stores. In return, two variables are included that refer to the different delivery policies of furniture online stores: the dummy variables *Dsco*_*j*_ and *Dscf*_*j*_ indicate whether delivery charges of online store *j* depend on the order value or whether they are free from a certain order value (which is offered by some furniture online retailers).

In addition, previous studies have, for the first time, examined whether omni-channel integration of retailers increases customer inflow and it was determined that with regard to consumer electronics and furniture retailers that this tends to be the case (Wieland 2021a, 2023). Thus, it is expected that retailers with an integrated online shop (see Sect. 3.3 for the definition) and which provide the “buy online, pick-up in store” (BIPOS) option will have a higher likelihood of consumer patronage. According to the previous studies, these two attributes of cross-channel integration are considered separately in the store utility function of the CE stores, using the dummy variables *DI*_*j*_ and *DC*_*j*_. The corresponding coefficients *γ*_*5*_ and *γ*_*6*_ in Eq. (4) are expected to be significant and positive. With furniture multi-channel retailers, it is the case that almost every store that has an integrated online shop also offers the BIPOS option. The two dummy variables would show almost perfect collinearity, so the dummies for the furniture stores are combined, with the variable *DCC*_*j*_ indicating whether store *j* has an integrated online shop *and* offers BIPOS (Wieland 2023). The corresponding coefficient *γ*_*6*_ in Eq. (5) is expected to be significant and positive.

To account for possible positive agglomeration effects, for consumer electronics stores, two variables reflect the potential for multipurpose shopping (dummy *DSM*_*j*_ for locations in a shopping mall) and comparison shopping (*C*_*j*_ for spatial proximity to competitors), respectively. Note that the cluster variable *C*_*j*_ is adapted from Fotheringham's (1985) Competing Destinations Model. If both types of agglomeration economies hold, then coefficients *γ*_*7*_ and *γ*_*8*_ in Eq. (4) would be significantly positive. German furniture stores are very rarely located in shopping malls, and in this case not a single furniture store is located within a mall. It is therefore pointless to include this independent variable in the store utility equation for furniture stores (see Eq. (5)).

Possible influences concerning membership of a specific store chain (e.g., *IKEA*, *Media Markt*) are not part of the research interest of this study, but it makes sense to take them into account in the model. This is made possible through dummy variables for the relevant chains: Dummy variable *D*_*gj*_ indicates whether store *j* belongs to chain *g*, and the corresponding coefficient *δ*_*g*_ displays the (positive or negative) chain effect. Additionally, to control for differences in the individual expenditures, the expenditure equation of the hurdle model contains the all-over expenditures of consumer *i*, *S*_*i*_.

The first part of the hurdle model explains the choice of (online or physical) shopping alternative *j*, in particular, the probability that the expenditures of consumer *i* at store *j* is greater than zero (*S*_*ij*_ > 0). This probability depends on the utility of shopping alternative *j*, ${V}_{ij}^{P}$ . The participation equation is formalized via a binary logit model:

6 $$Pr\left[{S}_{ij}>0|{V}_{ij}^{P}\right]=\mathrm{exp}\left({V}_{ij}^{P}\right)/\left(1+\mathrm{exp}\left({V}_{ij}^{P}\right)\right)$$

The second part of the hurdle model (intensity equation) deals with the amount of expenditure at the *chosen* stores (*S*_*ij*_ for all *S*_*ij*_ > 0). This part of the model is operationalized as a truncated Poisson distribution with a Poisson parameter of *λ*_*ij*_. The expected value depends on the store utility, ${V}_{ij}^{S}$:

7 $$E\left({S}_{ij},{S}_{ij}>0|{V}_{ij}^{S}\right)={\lambda }_{ij}/\left(1-{\mathrm{exp}\left(-{\lambda }_{ij}\right)}\right)$$

where

8 $$\mathrm{ln}{\lambda }_{ij}={V}_{ij}^{S}$$

The expected value of the store choice hurdle model (including both parts), *E(S*_*ij*_|*V*_*ij*_*)*, is:

9 $$E\left({S}_{ij}|{V}_{ij}\right)=\left(Pr\left[{S}_{ij}>0|{V}_{ij}^{P}\right]\right)\left(E\left[{S}_{ij},{S}_{ij}>0|{V}_{ij}^{S}\right]\right)$$

The estimation of the hurdle model employs the maximum likelihood technique (Greene 2012). The significance level is set to 90% (*p* < 0.1). Model estimation was conducted in *R* (R Core Team 2021) using the package *pscl* (Zeileis et al. 2008).

### Consumer survey

The empirical approach used in this study is analogous to previous research (Wieland 2021a, b, 2023). In order to answer the research questions, data on real-world shopping decisions as well as individual attitudes and socio-demographic characteristics was required. This data was collected in a self-administered postal consumer survey from September to December 2021 in three German planning regions (Regional Association of South Lower Saxony with 531,814 inhabitants, Middle Upper Rhine Region with 1,043,465 inhabitants, and Regional Planning Association Halle with 740,278 inhabitants). These study areas were chosen in order to incorporate both urban and rural regions (BBSR 2022) as well as both Western and Eastern Germany. In principle, the questionnaire was intended to be filled out using paper-and-pencil; however, the respondents were also given the option to fill it out in web form. The addresses of contacted individuals came from a random sample of the municipal registration offices, whereby the minimum age for making contact was set at 15 years. A total of 129 municipalities were asked in all three survey areas, of which 118 municipalities (91.5%) participated and provided address data.

In the questionnaire, shopping behavior was obtained by asking about purchases made in the recent past. The individuals were asked about their three last purchases of different goods (including consumer electronics and furniture) and the expenditures related to each purchase/shopping trip. For any purchase, the specific shopping destination (or online shop) was noted (e.g., “*Media Markt* in street *X* of municipality *Y*,” “*Amazon* online,” “*IKEA* online”). The expenditures of individual consumer *i* at (physical or online) store *j* is the dependent variable in the hurdle model (*S*_*ij*_). As an initial question, consumers were asked about their shopping frequency for specific goods.

The construction of the “pro online” attitude of consumer *i* (*PO*_*i*_) originally stems from the stated choice experiment by Schmid and Axhausen (2019). Their items related to risk perception for online shopping and attitudes toward in-store shopping were extended to include several statements connected with environmental and work-related effects of online shopping, as well as privacy issues (see Wieland 2021a, 2023 for a broader discussion). In the second part of the questionnaire, 15 attitude items on a 4-point Likert scale (1 = agree, …, 4 = disagree) were included. Two factors were extracted using an exploratory factor analysis (principal component extraction, Varimax rotation), one of which was expected to cover a “pro online” attitude.

The third part of the survey dealt with the pandemic situation from the respondent’s perspective. Fear of infection was targeted in order to examine any possible impact on shopping decisions. This section of the questionnaire included items from the *Perceived Vulnerability to Disease* (*PVD*) Scale (Duncan et al. 2009) in the same version (13 items) as used by Stangier et al. (2022). However, unlike this study, the PVD items were scaled on a 4-point Likert scale (1 = agree, …, 4 = disagree) to be congruent with the shopping attitude items. The two latent variables representing fear of infection (“perceived infectability,” “germ aversion”) were inferred using factor analysis in the same way as for shopping attitudes. One additional item on the same scale dealt with the perceived inconvenience of face masks in retail stores (“It bothers me to wear a mask while shopping”). The last part of the questionnaire asked for socio-demographic characteristics (age group, gender, employment status, and number of household members).

In the survey, 2,526 questionnaires were completed (see Table S1 in the online supplementary appendix). Considering neutral losses (e.g., invalid address, deceased), the response rate over the three survey regions was equal to 17.7%. There is a slight over-representation of women, whereas people from the lowest and the highest age group tended to be somewhat underrepresented. Nevertheless, this does not represent a problem as the model includes socio-demographic variables to control for such under- and overrepresentations. One-third of the respondents live in a large city (Göttingen, Karlsruhe, or Halle), as defined in the national classification system (BBSR 2022).

### Collection of store data

With respect to CE retailing, the inclusion criterion for consideration in the model analysis was that the respective (physical or online) store offered at least the following product range groups: “Electrical Household Appliances, Lighting (comprehensive)” *and* “Consumer Electronics, Electronic Media (comprehensive),” as defined by GfK (2020). Big-box stores (e.g., *Media Markt*, *Saturn*, *Euronics XXL*) typically also provide further GfK product range groups (“Information Technology,” “Telecommunications,” and “Photography, Optics [comprehensive]”). In order to distinguish between these and more specialized stores, the latter stores were marked with the dummy variable *Dnfr*_*j*_ (“not full range provider”) in the store choice model (see Sect. 3.1). Consequently, the collection of physical CE stores includes all big-box stores (e.g., *Media Markt*, *Saturn*, *Expert*) and specialty stores (e.g., *EP*), as well as some departments of department stores and hypermarkets (e.g., *Galeria*, *Real*). The same inclusion criterion was applied analogously to the online shops. The relevant online providers include, for example, the online shops of the omni-channel retail companies *Media Markt*, *Saturn*, and *Expert*, as well as pure online retailers with the corresponding range of products such as *Amazon* or *OTTO*. With respect to furniture shopping, all physical and online stores were included that offer the GfK ranges “Furnishings (comprehensive)” and “Household Products, Glass, Porcelain,” whereby a part of the range “Electrical Household Appliances, Lighting (comprehensive)” (especially lamps) is always covered (GfK 2020). The physical furniture stores considered therefore include the outlets of the chains of large stores such as *IKEA*, *XXXLutz*, or *Poco*. The online stores considered include the online shops of these chains as well as other purely online retailers such as *Amazon*.

For both physical and online stores, the number of articles offered (variable *A*_*j*_ in the store choice model) and information about their cross-channel integration were collected. The cross-channel integration of the chain or the specific store was ascertained via internet research, in particular, the specific online shop of the store/chain. “Cross-channel integration” is operationalized by two variables here, with the first indicating whether the specific store/chain has an “integrated online shop” (variable *DI*_*j*_) and the second indicating whether the “buy online, pick-up in store” (BOPIS) service is available (variable *DC*_*j*_). In the case of CE stores, both variables are included in the model. For the furniture stores, the variable *DCC*_*j*_ is included in the utility function that includes both attributes: *DCC*_*j*_ = *DI*_j_*DC_*j*_ (see Sect. 3.1). An “integrated online shop” was defined as the web platform of a multi-/cross-channel retailing company which provides information about (1) the assortment of both the online shop and the associated physical stores, *and* (2) the availability of each product in a given physical store, *and* (3) in-store price as well as some product details. The opportunity of BOPIS was verified simply by reviewing the company’s terms and conditions and the product pages. All online providers were researched for their delivery costs and their specified average delivery time (variables *sc*_*j*_ and *st*_*j*_). For all relevant physical stores, their location (street address) and store size (selling space in sqm) was recorded, with the latter being necessary for the interpolation of missing values on the assortment size (see below). Data on store size was requested from the corresponding companies and/or was obtained from public authorities, e.g., in land use plans.

The number of articles was quantified via desktop research using the websites and online shops of the corresponding retail companies (e.g., *Expert*, *IKEA*, *JYSK*, *Media Markt*). Most of the online shops of cross-channel retailers also provide an availability check for articles of each store, which allows the number of articles of specific stores of a chain to be counted. This procedure was automated using self-created functions and scripts for web scraping based on the package *httr* (Wickham 2019) in *R* (R Core Team 2021). While the number of items was completely available for all relevant CE online stores (27 in total), there was no data on assortment availability for 43 physical stores (108 CE stores in total). Therefore, the data of the stores for which both the selling space (in sqm) and the number of articles were available (49 stores) was used to estimate a regression model (see Table S2 in the online supplementary appendix) with which the number of articles was interpolated for the other stores. From a total of 123 physical furniture stores in all three study areas, the number of articles could be determined for 74 of them, and the sales area was also established for 50 stores. As with the CE stores, the missing values for the remaining 49 furniture stores were interpolated using an auxiliary regression (see Table S3 in the online supplementary appendix).

### Further data processing

The street addresses of the survey respondents (residential address) and physical stores were geocoded automatically by accessing the *OpenStreetMap* address database (*OSM Nominatim*). Based on these coordinates, travel times between consumer and store locations (variable *t*_*ij*_) were calculated using *OSRM* (*OpenStreetMap Routing Machine*), with travel time being defined as the fastest route between origins and destinations in terms of car driving time in minutes. It was assumed that car travel time is the best proxy for the opportunity costs of the shopping trip, since (1) it can be assumed that the majority of purchases at mostly decentralized CE and furniture stores are made by car, and (2) car travel times are estimated much more precisely than, e.g., public transport travel times. An interaction matrix for all *I* consumers (*i* = 1, …, *I*) and *J* stores (*j* = 1, …, *J*) with I*J rows was constructed and merged with the travel time matrix. The travel time variable *t*_*ij*_ was set to zero for online stores. The dependent variable *S*_*ij*_ (expenditures of consumer *i* at store *j*) was calculated from the survey data, whereby observed purchases without a record of the corresponding expenditures were excluded from the analysis. These steps were performed in *R* (R Core Team 2021) using the package *MCI2* (Wieland 2021c), which provides access to the OSM services by utilizing functions from the *OSRM* package for *R* (Giraud 2022).

The cluster variable *C*_*j*_ for the spatial proximity to competitors was calculated based on the street addresses of the stores. This was based on the airline distance between stores* j* and *k*, *d*_*jk*_, with the weighting exponent set to two. Dummy variables were also calculated for the individual chains. Both calculations mentioned were carried out in *R* using the *REAT* package (Wieland 2019).

Since the place of residence of the respondents was known, they could be assigned to the respective BBSR municipality type (BBSR 2022). This was necessary for the dummy variable *DL*_*i*_, which indicates whether a consumer lives in a large city (minimum of 100,000 inhabitants, as defined by the BBSR). It must be noted here that the division between “urban” and “rural” is of course relatively rough, especially since there are intermediate types in the spatial classification mentioned. However, the present analysis is not about differences in shopping behavior between region types in general, but very explicitly about the difference between (large) cities and (more or less) “rural” areas, which is discussed in the “innovation-diffusion hypothesis” (see Sect. 3.1). In the three survey areas, one can make a very clear distinction between the three “large cities” (Göttingen, Karlsruhe, Halle/Saale) and their (predominantly rural) surroundings. The next largest towns are many times smaller and the vast majority of surrounding communities in the three regions are “small towns” and “rural communities” (according to the aforementioned spatial type classification). In addition, the study aims (also) at a comparison with the time before the COVID-19 pandemic, whereby the results of previous studies (Wieland 2021a, 2023) are used for comparison. In these studies, a dummy variable was used to denote “large cities.” If in the present study the shopping behavior was broken down by other region types (which would in principle be more accurate), a comparison with the results from the pre-pandemic period would no longer be possible.

## Results and discussion

### Descriptive results toward shopping behavior

Tables 2 and 3 show descriptive results from the 2021 survey as compared to those of 2019 for each specific study area. Shopping frequencies stated by the respondents (median of CE or furniture purchases in the last 12 months), the shares of store formats and channels in the purchases and expenditures (in %), as well as the average travel times of the in-store purchases (car driving time in minutes, median) are displayed. Shares of CE and furniture purchases and expenditures separated by study area at the level of municipalities are shown in the online supplementary information (Figs. S3 and S4).

**Table 2:** Channel-specific purchases and expenditures by survey areas—Consumer electronics stores

	Purchases in numbers (%)		Expenditures in EUR (%)		Travel time in min (median)	
	2019	2021	2019	2021	2019	2021
*South Lower Saxony*						
Specialty store	5.7	7.0	15.9	6.1	9.7	6.8
Big-box store	48.2	44.2	49.9	73.5	16.5	14.2
Department	4.3	7.0	2.4	1.5	16.4	12.6
Online shop	41.9	41.9	31.8	18.8	–	–
Total	100.0	100.0	100.0	100.0	–	–
*Middle Upper Rhine Region*						
Specialty store	3.7	2.9	8.6	3.2	10.5	5.5
Big-box store	49.3	37.4	50.9	48.2	16.2	11.2
Department	3.7	4.1	2.5	1.5	14.2	9.4
Online shop	43.3	55.5	38.0	47.0	–	–
Total	100.0	100.0	100.0	100.0	–	–
*Regional Planning Association Halle*						
Specialty store	–	1.4	–	1.0	–	11.6
Big-box store	–	50.0	–	42.8	–	10.6
Department	–	2.9	–	1.2	–	12.1
Online shop	–	45.7	–	55.0	–	–
Total	–	100.0	–	100.0	–	–

Specialty stores include consumer electronics stores that sell at least the GfK product groups “Electrical Household Appliances, Lighting (comprehensive)” and “Consumer Electronics, Electronic Media (comprehensive)”; these include, above all, independent companies, most of which are affiliated with cooperatives (*EP*, *Euronics*, *Telering*). Big-box stores include the large-scale consumer electronics stores of *Expert*, *Euronics*, *Media Markt* and *Saturn*, while department stores include the consumer electronics departments of *Galeria Kaufhof/Karstadt* and some hypermarkets (*Edeka*, *Real*)

**Table 3:** Channel-specific purchases and expenditures by survey areas—Furniture stores

	Purchases in numbers (%)		Expenditures in EUR (%)		Travel time in min (median)	
	2019	2021	2019	2021	2019	2021
*South Lower Saxony*						
Physical stores	82.4	75.7	94.6	92.6	24.2	21.9
Online shop	17.6	24.3	5.4	7.4	–	–
Total	100.0	100.0	100.0	100.0	–	–
*Middle Upper Rhine Region*						
Physical stores	85.1	73.5	94.4	90.2	24.6	16.4
Online shop	14.9	26.5	5.6	9.8	–	–
Total	100.0	100.0	100.0	100.0	–	–
*Regional Planning Association Halle*						
Physical stores	–	62.3	–	86.1	–	21.7
Online shop	–	37.7	–	13.9	–	–
Total	–	100.0	–	100.0	–	–

If we look at the first two study areas, we can see that the frequency of consumer electronics and furniture purchases (measured in the form of the median) has remained the same. In this regard, there are no differences between the two or three study areas. It is noticeable that the average travel times for in-store consumer electronics purchases fell from 2019 to 2021 and that this applies to all three store formats considered. With respect to all physical stores of this industry, the decline is 21% (South Lower Saxony) and 31% (Middle Upper Rhine Region), respectively. A similar change can be seen in furniture purchases: the median travel times for purchases in physical stores fell by 9.3% in South Lower Saxony and by 33.2% in the Middle Upper Rhine Region. Even if this descriptive analysis precludes any causal conclusions, it is nevertheless an indication that there was less willingness to invest time in in-store shopping trips. A possible explanation for this could be that due to increased work-at-home during the pandemic, purchases on the way to work or at the place of work were reduced and instead purchases were made more frequently near the place of residence. In any case, it has now been proven that retail sales have shifted to the city suburbs during the pandemic, which is attributed to increased work-at-home frequency (Alipour et al. 2022).

In the first two study areas, the share of e-shopping in consumer electronics purchases is higher than in spending, which was already evident for 2019. This is also reflected in the average expenditures, which are higher in physical retailing than in online retailing. However, this does not apply to the third study area, where the share of expenditure is higher than the share of purchases and the median expenditure is nearly the same. The fact that expenditure in physical stores is higher on average than for online purchases can be seen with regard to furniture purchases in all three study areas. In line with expectations, the market share of e-commerce in consumer electronics and furniture retailing increased from 2019 to 2021, both in terms of purchases and expenditures. But the opposite picture emerges for consumer electronics purchases in the first study area. With regard to South Lower Saxony, it cannot be ruled out that the high share of expenditures for in-store consumer electronics shopping is due to “outliers” (some individual cases with extremely high purchase amounts) because the median expenditure in online stores is lower than in the Middle Upper Rhine Region. The online share of consumer electronics purchases is highest in the Middle Upper Rhine Region, the share of expenditures is highest in the Halle Region. Summarized across all three study areas, online retailing accounted for 46.4% of expenditure. For furniture purchases, online market share is highest for both purchases and expenditures in the third study area. In all cases, the share of purchases significantly exceeds the share of expenses since the average purchase amount is much lower for online purchases. Across all three study areas, the share of online expenditures equals 15.8%. Accordingly, online retailers play a noticeable role in shopping for consumer electronics and furniture products and the market share of online retailing has increased significantly.

### Psychographic consumer characteristics: shopping attitudes and fear of infection

Since shopping attitudes and fear of infection play an important role as (possible) explanatory variables in this study, it makes sense to take a closer look at these interim results. Table S4 in the online appendix shows the relative frequencies of the shopping attitude items, the associated latent variables, factor loadings, and additional information such as Cronbach’s α and cumulative explained variance. Table S5 in the online appendix shows the same for the PVD items.

Out of the 15 shopping attitude items, ten can be attributed to the first latent variable, the “pro online” attitude (variable *PO*_*i*_ in the model), whereas five items (1, 6, 8, 11–12) can be associated with the second latent variable, “physical shopping pleasure.” When interpreting the factor analysis, the scaling of the variables should be kept in mind (1 = “agree,” …, 4 = “disagree”), e.g., the *lower* the agreement with statement 2 (“Online shopping is associated with risks”), the *higher* the value of latent variable 1 (“pro online” attitude). Based on this relationship, the assignments to the latent variables are plausible in terms of content. The association of the variables with the factors, the cumulative explained variance and the internal consistency (Cronbach’s α), essentially correspond to the result of the first survey in 2019 (Wieland 2021a, 2023), and thus, also largely to that of Schmid and Axhausen (2019), from whose questionnaire the majority of the items was acquired. The value for Cronbach’s α for the “pro online” attitude equals 0.79, which can, at the very least, be classified as acceptable, but may also be rated as good. For these reasons, it can be assumed that the items used here sufficiently represent a “pro online” attitude.

The thirteen items on fear of infection are associated to the two latent variables (“perceived infectability” and “germ aversion”) as follows: Eight items are attributed to the first latent variable (2, 4–5, 7, 9, 11–13) and five items (1, 3, 6, 8, 10) to the second. The scaling of the items must be considered in the interpretation, e.g., the *higher* the agreement with statement 7 (“In general, I am very susceptible to colds, flu and other infectious diseases”), the *higher* the value of the “perceived infectability” factor (latent variable 1). It must be noted here that, analogous to the original paper by Duncan et al. (2009), some items were recoded (see Table S5 in the online appendix). This also applies to the item just mentioned, so that the highest numerical value corresponds to the *highest* level of agreement. Therefore, the factor loading of this item is positive (0.834). Against this background, the factor loadings can be regarded as understandable and plausible. The factor structure identified here is very similar to that found in the psychological studies from which the items are derived; the same applies to internal consistency and the explanation of variance (Duncan et al. 2009; Stangier et al. 2022). The first latent variable (“perceived infectability”) has good internal consistency and the second (“germ aversion”), at least, sufficient internal consistency. Since the results are very similar to those of psychological research, it is therefore assumed that the construct, “fear of infection,” is sufficiently represented to be operationalized for checking whether this characteristic influenced shopping behavior during the pandemic.

### Determinants of channel and store choice

For each of the three survey regions, one hurdle model consisting of two model parts was estimated. Tables 4 and 5 show the results with respect to CE and furniture shopping for the Middle Upper Rhine Region. Results for the other two survey areas can be found in the online appendix (Tables S6 to S9). The first column lists the explanatory variables, while the second column contains the coefficients of the Participation Equation (given in Eq. (6)), and the third column contains those of the Expenditure Equation (for all *S*_*ij*_ > 0, given in Eqs. (6)-(7)).^1^ In order to ensure the robustness of the models (e.g., with regard to possible collinearity effects), the estimation was carried out successively. Additional variables were gradually included in the models. This showed that the log-likelihood values could be increased and the results with regard to the significance of individual variables did not change (usually, changes in the coefficients only became apparent from the second decimal place). Correlations between individual independent variables where an association seemed possible were also checked; only weak correlations, if any, were found.^2^

**Table 4:** Estimation results for consumer electronics in the Middle Upper Rhine Region

Explanatory variables	2019		2021	
	Participation Equation	Expenditure Equation	Participation Equation	Expenditure Equation
**Store choice**				
*Store attraction*				
ln number of items_j_	0.968*** (0.074)	–0.083*** (0.003)	0.737*** (0.095)	–0.190*** (0.005)
Dummy MC with integrated online shop_j_	0.006 (0.371)	0.872*** (0.022)	–1.006** (0.511)	–1.513*** (0.019)
Dummy MC with BIPOS_j_	–0.298 (0.253)	–0.711*** (0.018)	0.066 (0.416)	0.628*** (0.015)
Dummy Saturn_j_	0.122 (0.336)	0.036** (0.016)	1.502*** (0.360)	0.900*** (0.014)
Dummy Media Markt_j_	–0.174 (0.332)	0.123*** (0.016)	1.822*** (0.352)	1.062*** (0.013)
Dummy Expert_j_	–0.601* (0.351)	0.235*** (0.017)	1.534*** (0.378)	1.851*** (0.014)
Dummy Euronics_j_	–0.800** (0.320)	0.230*** (0.014)	–0.989 (0.614)	1.323*** (0.026)
Dummy not full range_j_	–1.075*** (0.211)	0.105*** (0.011)	–0.212 (0.330)	–0.958*** (0.020)
Dummy located in shopping mall_j_	0.026 (0.111)	–0.084*** (0.006)	0.157 (0.144)	–0.048*** (0.006)
ln clustering_j_ + 0.0001	–0.063** (0.031)	0.011*** (0.002)	–0.118*** (0.037)	–0.015*** (0.002)
Dummy online store_j_	2.446** (1.045)	0.236*** (0.052)	0.520 (1.147)	1.130*** (0.047)
ln number of items_j_ x Dummy online store_j_	–0.743*** (0.085)	0.045*** (0.004)	–0.499*** (0.100)	0.029*** (0.005)
*Shopping transaction costs*				
Travel time_ij_	–0.171*** (0.006)	0.002*** (0.0003)	–0.185*** (0.008)	0.040*** (0.0003)
Delivery time_j_	–0.487*** (0.120)	–0.028*** (0.003)	–0.444*** (0.078)	–0.003 (0.002)
Delivery charges_j_	–0.340*** (0.051)	–0.010*** (0.002)	–0.172*** (0.029)	–0.170*** (0.001)
**Channel choice**				
*Socio-demographic and spatial consumer attributes*				
Dummy place of residence is large city_i_	–1.454*** (0.119)	–	–1.041*** (0.142)	–
Dummy online store_j_ x Dummy place of residence is large city_i_	1.363*** (0.171)	–	1.132*** (0.180)	–
Dummy age < 25_i_	0.376** (0.154)	–	0.571** (0.250)	–
Dummy age > = 65_i_	0.138 (0.160)	–	0.264 (0.216)	–
Dummy male_i_	0.234** (0.093)	–	0.388*** (0.122)	–
Dummy employed_i_	0.234* (0.131)	–	–0.056 (0.196)	–
Dummy online store_j_ x Dummy age < 25_i_	–0.336 (0.239)	–	–0.764** (0.338)	–
Dummy online store_j_ x Dummy age > = 65_i_	–1.526*** (0.301)	–	–1.016*** (0.294)	–
Dummy online store_j_ x Dummy male_i_	0.115 (0.150)	–	–0.305* (0.161)	–
Dummy online store_j_ x Dummy employed_i_	–0.218 (0.202)	–	0.079 (0.246)	–
*Shopping attitudes*				
LV pro online_i_	–0.173*** (0.050)	–	–0.260*** (0.065)	–
Dummy online store_j_ x LV pro online_i_	0.578*** (0.080)	–	0.558*** (0.086)	–
*Attitudes toward pandemic situation*				
LV perceived infectability_i_	–	–	–0.119* (0.062)	–
LV germ aversion_i_	–	–	0.104* (0.061)	–
Face mask aversion_i_	–	–	0.052 (0.050)	–
Dummy online store_j_ x LV perceived infectability_i_	–	–	0.197** (0.080)	–
Dummy online store_j_ x LV germ aversion_i_	–	–	–0.112 (0.080)	–
Dummy online store_j_ x Face mask aversion_i_	–	–	–0.119* (0.067)	–
ln expenditures_i_	–	0.837*** (0.002)	–	0.868*** (0.001)
Constant	–6.910*** (0.616)	0.809*** (0.028)	–6.601*** (0.851)	1.457*** (0.040)
Observations	49,630		34,643	
Log likelihood	–94,076.37		–159,875.20 (B)	
AIC	188,242.70		319,860.50 (B)	

The Participation Equation relates to Eq. (6), while the Expenditure Equation relates to Eq. (7). * p < 0.1; ** p < 0.05; *** p < 0.01. Coefficient standard errors in parentheses. MC = Multi-channel retailer, LV = latent variable. Data for 2019 from Wieland (2021a)

**Table 5:** Estimation results for furniture in the Middle Upper Rhine Region

Explanatory variables	2019		2021	
	Participation equation	Expenditure equation	Participation equation	Expenditure equation
**Store choice**				
*Store attraction*				
ln number of items_j_	0.902*** (0.074)	–0.016*** (0.002)	0.712*** (0.100)	–0.081*** (0.002)
Dummy Cross-Channel Retailer_j_	1.315*** (0.383)	0.045*** (0.007)	0.449** (0.203)	0.179*** (0.004)
Dummy JYSK_j_	–1.560*** (0.437)	–0.936*** (0.012)	–0.153 (0.382)	–1.388*** (0.015)
Dummy IKEA_j_	2.022*** (0.429)	–0.627*** (0.009)	1.901*** (0.181)	–0.625*** (0.005)
Dummy Poco_j_	––3.173*** (0.705)	–0.892*** (0.047)	–	–
Dummy Mömax_j_	–0.838* (0.434)	-0.422*** (0.010)	0.975*** (0.258)	-0.094*** (0.005)
Dummy Roller_j_	–2.496*** (0.457)	-0.372*** (0.011)	-1.291*** (0.309)	-0.556*** (0.012)
Dummy XXXLutz_j_	–1.537*** (0.429)	–0.028*** (0.009)	0.338 (0.228)	0.202*** (0.005)
Dummy Amazon_j_	1.062** (0.431)	0.681*** (0.027)	0.899** (0.411)	0.246*** (0.028)
Dummy eBay_j_	0.266 (0.463)	1.170*** (0.027)	0.679 (0.441)	0.518*** (0.031)
ln clustering_j_ + 0.0001	0.036 (0.029)	–0.015*** (0.001)	0.070*** (0.020)	–0.053*** (0.001)
Dummy online store_j_	3.441*** (0.988)	0.468*** (0.033)	5.859*** (1.292)	–3.688*** (0.067)
ln number of items_j_ x Dummy online store_j_	––0.796*** (0.086)	–0.141*** (0.003)	–0.606*** (0.118)	0.125*** (0.005)
*Shopping transaction costs*				
Travel time_ij_	–0.102*** (0.004)	0.002*** (0.0001)	–0.073*** (0.005)	–0.002*** (0.0001)
Delivery charges_j_	–0.009 (0.007)	0.008*** (0.0004)	–0.018** (0.008)	0.019*** (0.0005)
Dummy delivery charges based on order value_j_	–1.200*** (0.356)	0.113*** (0.018)	–0.048 (0.319)	0.965*** (0.015)
Dummy free delivery from a certain order value_j_	–1.008*** (0.340)	–0.284*** (0.016)	–1.222*** (0.444)	–0.972*** (0.029)
**Channel choice**				
*Socio-demographic and spatial consumer attributes*				
Dummy place of residence is large city_i_	–0.519*** (0.095)	–	–0.575*** (0.124)	–
Dummy online store_j_ x Dummy place of residence is large city_i_	0.535** (0.212)	–	0.693*** (0.221)	–
Dummy age < 25_i_	–0.071 (0.163)	–	0.299 (0.244)	–
Dummy age > = 65_i_	0.024 (0.158)	–	0.174 (0.216)	–
Dummy male_i_	0.026 (0.090)	–	0.204* (0.117)	–
Dummy employed_i_	0.103 (0.124)	–	0.121 (0.182)	–
Dummy online store_j_ x Dummy age < 25_i_	0.520* (0.295)	–	–0.625 (0.464)	–
Dummy online store_j_ x Dummy age > = 65_i_	–1.799*** (0.563)	–	–1.659*** (0.499)	–
Dummy online store_j_ x Dummy male_i_	–0.290 (0.207)	–	–0.442** (0.219)	–
Dummy online store_j_ x Dummy employed_i_	–0.279 (0.255)	–	–0.287 (0.308)	–
*Shopping attitudes*				
LV pro online_i_	–0.019 (0.047)	–	–0.132** (0.061)	–
Dummy online store_j_ x LV pro online_i_	0.320*** (0.108)	–	0.420*** (0.114)	–
*Attitudes toward pandemic situation*				
LV perceived infectability_i_	–	–	0.025 (0.055)	–
LV germ aversion_i_	–	–	0.027 (0.055)	–
Face mask aversion_i_	–	–	0.068 (0.047)	–
Dummy online store_j_ x LV perceived infectability_i_	–	–	–0.086 (0.103)	–
Dummy online store_j_ x LV germ aversion_i_	–	–	–0.098 (0.102)	–
Dummy online store_j_ x Face mask aversion_i_	–	–	–0.155* (0.091)	–
ln expenditures_i_	–	0.848*** (0.001)	–	0.892*** (0.001)
Constant	–8.693*** (0.662)	0.969*** (0.019)	–10.024*** (0.897)	1.692*** (0.018)
Observations	47,877		29,421	
Log likelihood	–207,738.10		–157,415.600	
AIC	415,574.10		314,937.20	

The Participation Equation relates to Eq. (6), while the Expenditure Equation relates to Eq. (7). * p < 0.1; ** p < 0.05; *** p < 0.01. Coefficient standard errors in parentheses. LV = latent variable. Data for 2019 from Wieland (2023)

First, the results related to the choice of shopping channel (channel utility, ${V}_{ij}^{C}$) are considered, which represent one part of the participation equation. The innovation-diffusion hypothesis states that urban residents are more likely to buy online, which has been confirmed for South Lower Saxony and the Middle Upper Rhine Region with respect to consumer electronics and furniture purchases in 2019 (see the participation equations for 2019). However, with respect to consumer electronics purchases, the coefficient for the interaction term, *DO*_j_*DL_*i*_, *β*_*15*_, for the 2021 data is only significantly positive in the second study area, but not (anymore) in South Lower Saxony and Halle. Regarding the models for furniture shopping, the associated coefficient is also only significantly positive in the second study area. Therefore, the assumption that people in large cities tend to shop more online can only be confirmed for one of the three study areas. It is in any case questionable whether such a result can confirm the innovation-diffusion hypothesis at all. On the one hand, it is questionable whether e-shopping can still be considered an “innovation,” given that online shopping has been available for over 20 years. On the other hand, a tendency toward more online purchases in (large) cities, especially for durable goods, can also be explained differently. Big-box stores for durable goods such as consumer electronics and furniture are often located in peripheral commercial areas and the car density is significantly lower in large cities. Therefore, the accessibility of such stores in large cities is sometimes poor, which would rather speak for a confirmation of the efficiency hypothesis (Wieland 2021a, 2023).

There is a clear result regarding shopping attitudes. The coefficient of the interaction term, *DO*_j_*PO_*i*_, *β*_*16*_, is significant and positive in all three regions for consumer electronics purchases and in two of three study areas for furniture purchases (the coefficient is positive but not significant in South Lower Saxony). This result was already found in 2019 in the first two study areas and is now confirmed for the Middle Upper Rhine Region and the Halle Region; it is also congruent with the experimental choice study by Schmid and Axhausen (2019) and real-world shopping behavior with respect to groceries (Wieland 2021b). Channel choice may, therefore, be regarded as highly impacted by attitudes toward the online channel, which only seems obvious at first glance. It is by no means to be assumed that stated attitudes (e.g., regarding the impact of online shopping on the environment or on working conditions) are congruent with actual behavior; this may be due, among other things, to the social desirability bias in surveys. The result is also important because the innovation-diffusion hypothesis relates to psychographic consumer characteristics, implying that online affinity is greater in urban areas. On the contrary, we find, in the current study, predominantly no influence of the place of residence, but of the attitude itself. Within the scope of this study, it cannot be clarified why the “pro online” attitude in the first study area (South Lower Saxony) no longer plays a statistically demonstrable role in channel choice for furniture purchases.

It was assumed that shopping online rather than in-store could be a strategy to reduce one's risk of infection. The coefficients for the two interaction terms of the online dummy with the latent variables “perceived infectability” and “germ aversion,” *β*_*17*_ and *β*_*18*_, are typically found to be not significantly positive. There are only two exceptions. First, in the Middle Upper Rhine Region is an increasing degree of perceived infectability associated with a higher probability of buying consumer electronics online. Fear of infection (as operationalized here using the psychological PVD scale) therefore plays little or no role in channel choice for consumer electronics purchases, at least in this survey from 2021. Second, a significant effect of germ aversion in channel choice for furniture purchases was found in the first study region (South Lower Saxony). There was no significant effect of perceived infectability in any of the three areas. The fact that neither of the two latent variables that represent fear of infection significantly influences channel choice is the norm with regard to the two product types considered and the three regions examined. To some extent, these results contradict those of Stangier et al. (2022), who found fear of infection as a predictor for (declared) changes in behavior (in terms of risk avoidance, etc.). It must also be taken into account that such attitudes may have changed; the work of Stangier et al. (2022) refers to the first year of the pandemic, whereas the results shown here concern shopping behavior in the second year. The same applies to studies that dealt with channel choice in the early days of the pandemic and found influences of fear of infection and perceived insecurity (e.g., Chenarides et al. 2021).

There is no indication that the rejection of masks in shops influences the choice of shopping channel in favor of online retailing, as the related coefficient, *β*_*19*_, is not significant and positive for either product type considered in any of the three study areas. With respect to CE purchases, in two out of three study areas there is instead a significantly negative coefficient, which contradicts the expected effect. The same applies to furniture purchases in the second survey area. The reason for this may lie in unobserved consumer characteristics (e.g., with regard to lifestyle).

As expected, a pronounced age effect can be determined for the socio-demographic control variables. Older consumers (65 years and older) shop significantly less online, which is reflected in most models. At the same time, it has also been partially shown that young consumers (under 25 years) shop online significantly more often. The findings of the most models also demonstrate that male consumers shop online significantly less often, which is not consistent with the results of previous studies. With regard to the employment status, no clear statements can be made as the associated interaction variable in the CE models is only significant in one study area and in the case of furniture purchases the results contradict each other (positive in the first region, not significant in the second, negative in the third).

With respect to store utility, the impact of assortment is found to have a significant impact, as the coefficient γ_1_ of *ln A*_*j*_ is significant in both model parts in all three survey areas for both consumer electronics and furniture shopping. This influence, however, is not uniform. In the Participation Equations for consumer electronics, which reflect store choice probability, the coefficient is between zero and one in the first two survey areas but negative in the third region. In the case of the Participation Equation for furniture purchases, the associated coefficient is within this range in all three study regions. A coefficient between zero and one implies a sublinear positive impact of assortment on consumer utility, as stated, e.g., in the Huff model (Huff 1962). The positive effect is smaller for online stores as the coefficient γ_10_ of the interaction term, *ln A*_j_*DO_*j*_ is significant and negative in the first two study regions. This may be explained by the fact that online shops have a much larger assortment (in terms of breadth and depth of product range), implying that expanding this range leads to a much smaller increase in consumer utility. With respect to South Lower Saxony and the Middle Upper Rhine Region, the results from the 2019 survey were confirmed. Comparable results can also be seen with respect to furniture shopping (Wieland 2023). However, the results with respect to CE shopping for the Regional Planning Association Halle are the opposite. This may be due to a fundamentally different retail structure and, related to this, different consumer needs in the Halle Region (representing East Germany). The CE market there is dominated by peripherally located big-box stores with a large assortment, and the share of expenditure in e-shopping is, for this area, by far the highest. In this competitive environment, product range extensions may no longer play a role in shopping decisions.

The results concerning the shopping transaction costs are very clear, at least assuming consumers strive to reduce these costs as much as possible (Chintagunta et al. 2012). For both consumer electronics and furniture purchases and in all three survey regions, the coefficient γ_2_ of travel time, *t*_*ij*_ is significant and negative in the Participation Equation, which means that the greater the travel time to the (physical) store, the lower its choice probability. Distance-dependent demand or reduction in consumer utility due to opportunity costs of travel is one of the fundamental statements of retail location theory (e.g., Christaller 1933; Huff 1962) and has been empirically confirmed countless times (see Sect. 2.1). The same applies analogously to the shopping transaction costs for online retailers, at least regarding the models for CE shopping. The coefficients of delivery costs, γ_3_, and delivery time, γ_4_, are significant and negative in the Participation Equations in all three study regions. While delivery costs represent real expenses, delivery time can also be interpreted as the opportunity cost of waiting. In the case of furniture shopping, however, this expectation can only be partially confirmed, as the delivery charges only show the expected significant negative effect in one of the three study areas (Middle Upper Rhine Region). In the 2019 survey, delivery charges in the furniture retail sector were of no significant importance. This might be explained by the fact that the high expenses incurred when ordering furniture means that shipping costs are no longer a decisive factor for consumers. Other aspects of the delivery policy of furniture online stores influence store choice, which is shown by the fact that the dummy variables *Dsco*_*j*_ and *Dscf*_*j*_ sometimes have significant influences. These impacts are not uniform across the study regions and are not the focus here.

In summary, it can be said that in the case of store choice, the transaction costs of the channel or the individual store regularly reduce consumer utility and choice probability, respectively. However, in the intensity equation, these costs do not always show a negative, but sometimes a positive effect. This seems contradictory at a first glance, but it is possible that consumers compensate for the increased effort (e.g., longer travel time, higher delivery costs) by making a larger purchase. The results regarding the shopping transaction costs largely confirm the findings from the 2019 survey.

With regard to CE purchases, a clear difference compared to the results on shopping behavior before the pandemic can be seen in the influence of the cross-channel integration of the stores. In contrast to the previous model-based analysis, there is no longer a positive effect of an integrated online shop or the “buy online, pick-up in store” (BOPIS) option, since the two relevant coefficients, γ_5_ and γ_6,_ for the dummy variables *DI*_*j*_ and *DC*_*j,*_ are usually either insignificant or significantly negative. This applies without exception to the participation equations, which means that the cross-channel integration of CE retailers does *not* increase consumer utility and choice probability, respectively.

In the furniture retail sector, the result is similar, with one exception. In the first study area (South Lower Saxony), a significantly positive effect was found for the cross-channel integration of a store/chain in the 2019 survey, which can now no longer be confirmed because the associated coefficient γ_6_ of the variable dummy variable *DCC*_*j*_ is no longer significant. Likewise, no such influence on store choice can be found in the Halle region, while the utility-enhancing effect of cross-channel integration continues to be confirmed in the Middle Upper Rhine Region. As a result, no increase in the consumer utility of a store through its cross-channel integration can regularly be determined. One possible reason for this are changes occurring in the retail landscape, whereby some physical stores have, on the one hand, set up additional online shops and, on the other, some companies without any cross-channel integration have left the market.

In addition, the enormously increased relevance of online shopping is easier for large chains to cope with when compared to non-chain stores. It is therefore possible that integrated online shops and BIPOS, at least in consumer electronics and furniture retailing, have become so common that they no longer represent a competitive advantage. This especially appears plausible in the context of the COVID-19 pandemic, where many physical retailers started to engage in multi-channel retailing. Thus, the declining importance of cross-channel integration as a competitive advantage would also be an indirect consequence of the pandemic situation and the governmental interventions associated with it.

Positive agglomeration effects due to the potential of multipurpose and comparison shopping cannot be detected in all but one case, as the corresponding coefficients of the variables *C*_*j*_ and *DSM*_*j*_ (the latter being tested only in the case of CE purchases) are mostly insignificant, and in some cases, negative. However, the clustering variable has a significantly positive influence on store choice for furniture purchases in the second study area (Middle Upper Rhine Region). This result is unique and appears to be contradictory at first glance. It is most likely due to differences in the location structure of the furniture stores between the study areas. A negative effect indicates the clustering of competitors whose assortments tend to be substitutable. On the other hand, a positive effect, as in the Middle Upper Rhine, suggests a cumulative attraction of stores (Nelson 1958). For example, there is a furniture cluster in the city of Karlsruhe in survey area 2, which includes an *IKEA* store that opened in 2020 and stores of two other chains. Since this effect was not yet found in the 2019 survey in the same area and the *IKEA* opening represents the only change in the sector under consideration, it strongly suggests that the *IKEA* settlement made this cluster effect possible. There is empirical evidence for positive agglomeration effects arising from *IKEA* openings (Daunfeldt et al. 2019). It is also important to note that clustering increases the choice probability in the mentioned case but not the expenditure, since γ_7_ in the intensity equation is negative. This indicates that while there is a cumulative attraction, the actual expenditure is split between the competitors.

There are also several significant chain effects with respect to both product types considered (e.g., regarding *IKEA*, *Media Markt* and *Saturn*) which are most likely largely determined by the competitive structure of the respective study region, and which are outside the scope of this study. The control variable for total expenditures by consumer correlates, as expected, positively with the expenditures in the stores.

## Conclusions and limitations

*First*, it is important to conclude that the competitive relationships between online and physical retailers can be incorporated into a store choice model for spatial shopping behavior. With regard to the assumptions concerning consumer behavior (e.g., reduction of transaction costs, accessibility), there are large overlaps between traditional retail location theory (e.g., Huff model, CPT) and newer concepts around multi-channel shopping behavior (e.g., transaction costs perspective, efficiency hypothesis). As the models presented here show, these concepts can be combined, which represents an advance of the theoretical and methodological concepts of retail geography. Another advantage of the model is that it works at the individual level meaning that individual characteristics of both consumers and stores can be considered. From a practical perspective, for the purpose of business expansion or estimating the impact of new retail projects in urban and regional planning, it makes sense to take online retailing into account. The store choice model developed here provides a possible approach to this.

*Second*, the determinants of shopping behavior have essentially not changed compared to the period before the pandemic. The preference for online shopping can be explained by psychographic consumer attributes, more precisely, by shopping attitudes toward the online channel, age of the consumers, and, partially, place of residence. The choice of the specific (physical or online) store is determined primarily by shopping transaction costs occurring during the purchase (travel time, delivery time, delivery costs), and store features such as assortment. Several substantial differences in spatial shopping behavior before the pandemic (2019) and during the pandemic (2021) have been identified, such as the decline of the average travel time for in-store purchases or the lack of cross-channel integration as a competitive advantage. These changes in shopping behavior mentioned can be regarded as *indirect* consequences of the COVID-19 pandemic.

*Third*, with respect to *direct* consequences of the pandemic situation, the results indicate that fear of infection and the obligation to wear a mask have no or only a very minor influence on channel choice. At least it cannot be generally stated that consumers would have avoided in-store shopping and shopped online instead because of fear of infection (in retail stores), or because of a rejection of face masks. The pandemic situation was therefore—at least during the period under review—not a key driver of competition between the shopping channels. This does not mean that this influence did not exist in the first year of the pandemic; it must be emphasized that the purchases examined took place in 2021, at a time when the COVID-19 vaccination was already available and a large part of the population was vaccinated.

Despite these important findings, the current study also has limitations. *First*, there is a concern regarding the econometric strategy. Although the model approach includes and distinguishes between channel choice and store choice, it does not reveal the order in which these two consumer decisions are made. For example, it is possible for the channel to be selected first and then the store to be selected. Another decision-making level may also be interposed with respect to the store format (big-box store, specialty store etc.). In order to find out whether the decision is made sequentially or simultaneously, more differentiated surveys and models are necessary. One way to depict a hierarchical decision would be a nested logit model (e.g., Suel and Polak 2017), but this type of model is also based on mathematical considerations regarding the substitution of alternatives and cannot directly show the order in which the decisions are actually made. In addition, in such a model only the decision is modeled and not, as in the hurdle model, also the expenditure; therefore this would not be a satisfactory alternative.

*Second*, with regard to the informative value of the model results, it should also be considered that part of the empirically recorded purchases had to be excluded (e.g., purchase directly from the manufacturer) in order to ensure comparability of the providers included within the model. Of course, this means that information is lost.

*Third*, the underlying consumer survey was conducted from September to December 2021, which means that the recorded purchases were made in the previous months. Thus, the empirical data used here relate to shopping decisions outside the peak times of SARS-CoV-2 infections (which was also deliberately integrated into the study design as there were no or few other Corona-related restrictions on retailing during this time). However, it is quite possible that during the peak of the infection waves—for example, in December or January—the effect of fear of infection on shopping behavior was significantly greater. In addition, it can also be assumed that fear of infection played a far greater role in the first few months of the pandemic (especially spring 2020) than in the second year of the pandemic. The results shown here do not allow this conclusion to be ruled out. Furthermore, in winter 2021/2022, i.e., following the purchases recorded here, there were new restrictions for retail stores (e.g., access restrictions for the unvaccinated), which were not able to be considered here.

*Fourth*, it must be remembered that the modeling approach used here operates entirely at the individual level, i.e., the individual consumer (and where they live) and the individual store. On the one hand, this is an advantage because there are no biases due to spatial aggregations. On the other hand, the modeling approach does not allow any statements about differences in shopping behavior between region types or intra-regional differences (i.e., *within* the three survey regions). The only difference considered in this study is that between (large) cities and rural areas, whereby this distinction is not made at a spatial level, but at the consumer level. In order to take the spatial scale more into account, either separate models for each region type or a large number of other interaction variables would be necessary, which is not done at this point.

## Supplementary Information

Below is the link to the electronic supplementary material.

Supplementary file1 (PDF 1500 KB)

## Footnotes

1 The results of the 2019 survey are also shown for the first two survey areas.

2 Two variants of the participation equation were estimated, one without the pandemic-related psychographic variables and one with. There was no relevant change in the other variables or coefficients. However, the models that contain the Corona variables are characterized by a slightly higher log-likelihood value and a slightly lower AIC value, which is why they are used for the following interpretation.
