# Supplementary material for: Spatial shopping behavior during the Corona pandemic: insights from a micro-econometric store choice model for consumer electronics and furniture retailing in Germany
Source: J Geogr Syst. 2023 Apr 3;25(2):291–326. doi: 10.1007/s10109-023-00408-x (PMC10069365; doi:10.1007/s10109-023-00408-x)
Supplement: Supplementary file 1 — Supplementary file1 (PDF 1500 KB) [file 10109_2023_408_MOESM1_ESM.pdf]

Title:
Spatial shopping behavior during the Corona pandemic: Insights from a micro-econometric store choice model for consumer electronics and furniture retailing in Germany

In:
Journal of Geographical Systems

Corresponding author:
Thomas Wieland

Author information:
Dr. Thomas Wieland
Karlsruhe Institute of Technology (KIT)
Institute of Geography and Geoecology, Human Geography
Kaiserstr. 12
76131 Karlsruhe
Germany
E-Mail: Thomas.Wieland@kit.edu
ORCiD: https://orcid.org/0000-0001-5168-9846

SUPPLEMENTARY INFORMATION

Content

Table S1: Socio-demographic characteristics of the respondents. 1
Table S2: Consumer electronics stores – Regression model for interpolation of assortment (Dependent variable: ln number of articles). 2
Figure S1: Consumer electronics stores – Correlation between store size and number of articles. 3
Table S3: Furniture stores - Regression model for interpolation of assortment (Dependent variable: ln number of articles). 4
Figure S2: Furniture stores – Correlation between store size and number of articles. 5
Figure S3: Consumer electronics purchases and expenditures separated by study area at the municipality level. . 6
Figure S4: Furniture purchases and expenditures separated by study area at the municipality level. 7
Table S4: Relative frequencies and factor loadings of attitude items (non-missing only). 8
Table S5: Relative frequencies and factor loadings of the Perceived Vulnerability to Disease (PVD) scale (non-missing only). 9
Table S6: Consumer electronics stores – Modeling results for survey area 1 (South Lower Saxony). 10
Table S7: Consumer electronics stores – Modeling results for survey area 3 (Regional planning association Halle). 12
Table S8: Furniture stores – Modeling results for survey area 1 (South Lower Saxony). 14
Table S9: Furniture stores – Modeling results for survey area 3 (Regional planning association Halle). 16
References 18

Table S1: Socio-demographic characteristics of the respondents.

Criterion		N [no.]	N [% of 2,526]
Age category [years]	15 – 17	19	0.8
	18 – 24	109	4.3
	25 – 44	412	16.3
	45 – 64	950	37.6
	65 – 74	554	21.9
	75 and older	414	16.4
	Missing	68	2.7
Gender	Female	1,413	55.9
	Male	1,038	41.4
	Divers	9	0.4
	Missing	66	2.6
Household size [persons]	1	454	17.9
	2	1,242	49.2
	3	360	14.3
	4	262	10.3
	> 4	118	4.7
	Missing	90	3.5
Employment status	Employed or self-employed	1,130	44.7
	Retired	1,069	42.3
	School or university	100	4.0
	Not employed (homemaker m/f)	49	1.9
	Unemployed	36	1.4
	Other	34	1.3
	Missing	108	4.3
Place of residence	Large city	834	33.0
	Other	1,682	66.6
	Missing	10	0.4
Study area	South Lower Saxony	391	15.5
	Middle Upper Rhine Region	1,261	49.9
	Halle Region	866	34.4
	Missing	8	0.3
Type of survey	Written survey (mail)	2,315	91.6
	Online survey	211	8.4

Note: Because of missing values, the sample sizes differ for each characteristic.

Table S2: Consumer electronics stores – Regression model for interpolation of assortment (Dependent variable: ln number of articles).

**Explanatory variables**	**Coefficients (std. errors)**
ln store size$_j$	0.372**<br>(0.175)
Dummy Saturn$_j$	1.983***<br>(0.706)
Dummy Media Markt$_j$	1.816**<br>(0.694)
Dummy Expert$_j$	1.736***<br>(0.603)
Dummy Euronics$_j$	1.126*<br>(0.570)
Dummy Electronic Partner$_j$	–1.096***<br>(0.305)
Dummy Medimax$_j$	0.068<br>(0.586)
Constant	4.976***<br>(0.762)
Observations	49
R^2	0.921
Adjusted R^2	0.907
Residual Std. Error	0.512 (df = 41)
F Statistic	68.073*** (df = 7; 41)

Note: * p < 0.1; ** p < 0.05; *** p < 0.01

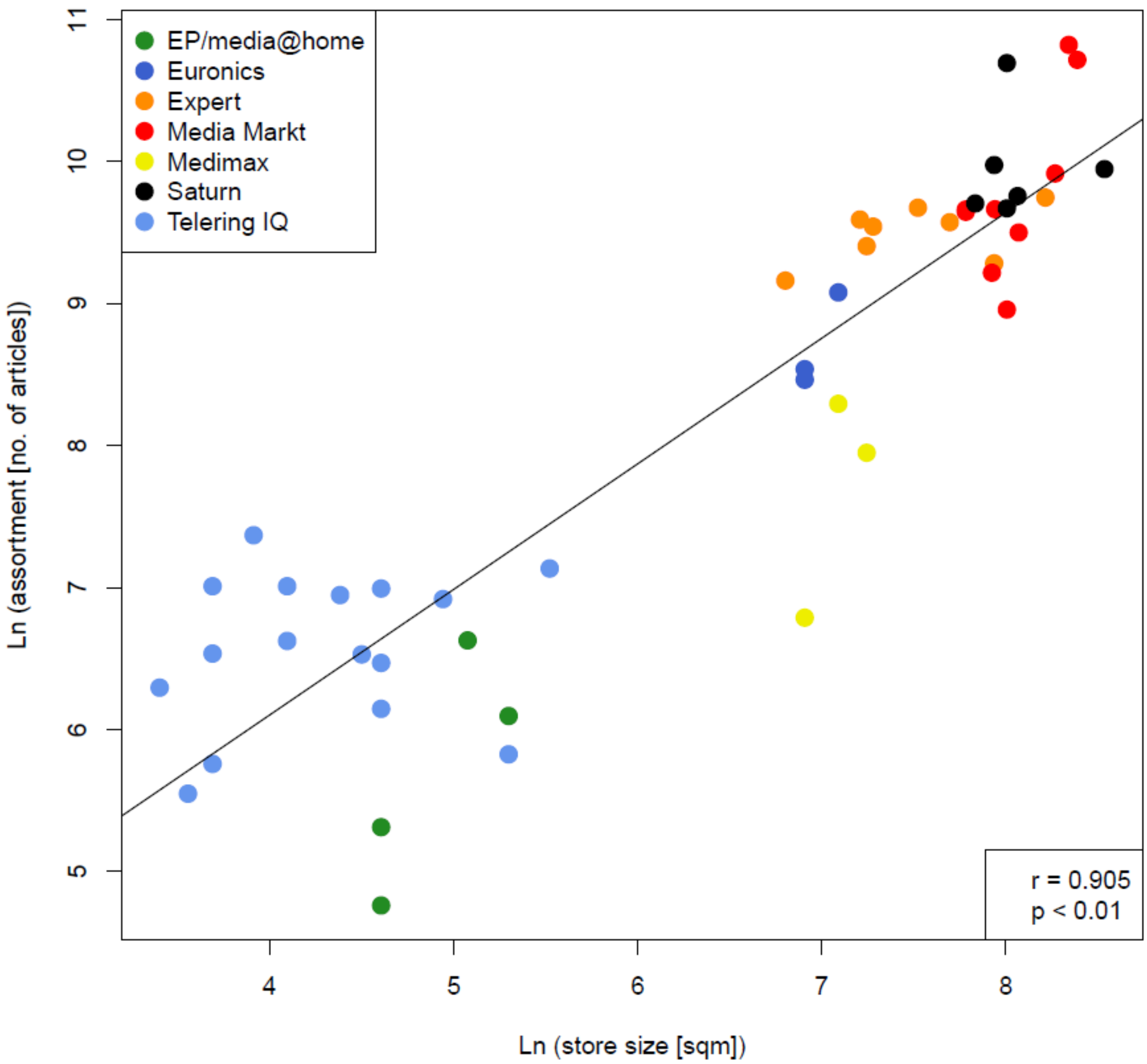

Figure S1: Consumer electronics stores – Correlation between store size and number of articles.

Table S3: Furniture stores - Regression model for interpolation of assortment (Dependent variable: ln number of articles).

**Explanatory variables**	**Coefficients (std. errors)**
ln store size$_j$	1.442*** (0.180)
Dummy IKEA$_j$	0.067 (0.300)
Dummy JYSK$_j$	2.002*** (0.482)
Dummy Moebel Hoeffner$_j$	1.397** (0.639)
Dummy Moemax$_j$	–0.110 (0.363)
Dummy Poco$_j$	1.307*** (0.366)
Dummy Porta$_j$	–0.917 (0.629)
Dummy Roller$_j$	1.615*** (0.362)
Dummy SB Moebel Boss$_j$	0.240 (0.384)
Dummy XXXLutz$_j$	0.103 (0.508)
Constant	–4.689*** (1.638)
Observations	50
R^2	0.890
Adjusted R^2	0.861
Residual Std. Error	0.556 (df = 39)
F Statistic	31.416*** (df = 10; 39)

Note: * p < 0.1; ** p < 0.05; *** p < 0.01

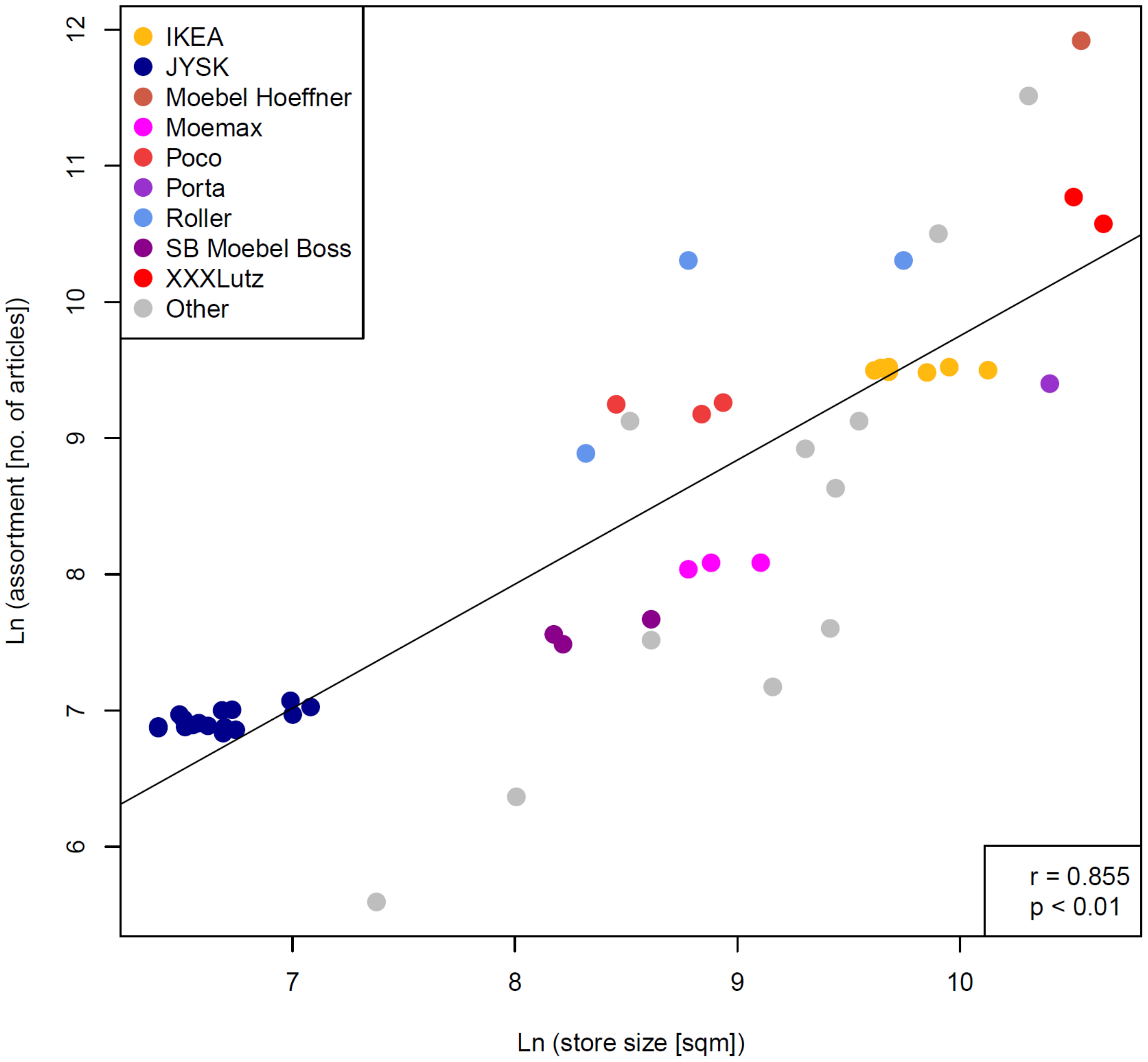

Figure S2: Furniture stores – Correlation between store size and number of articles.

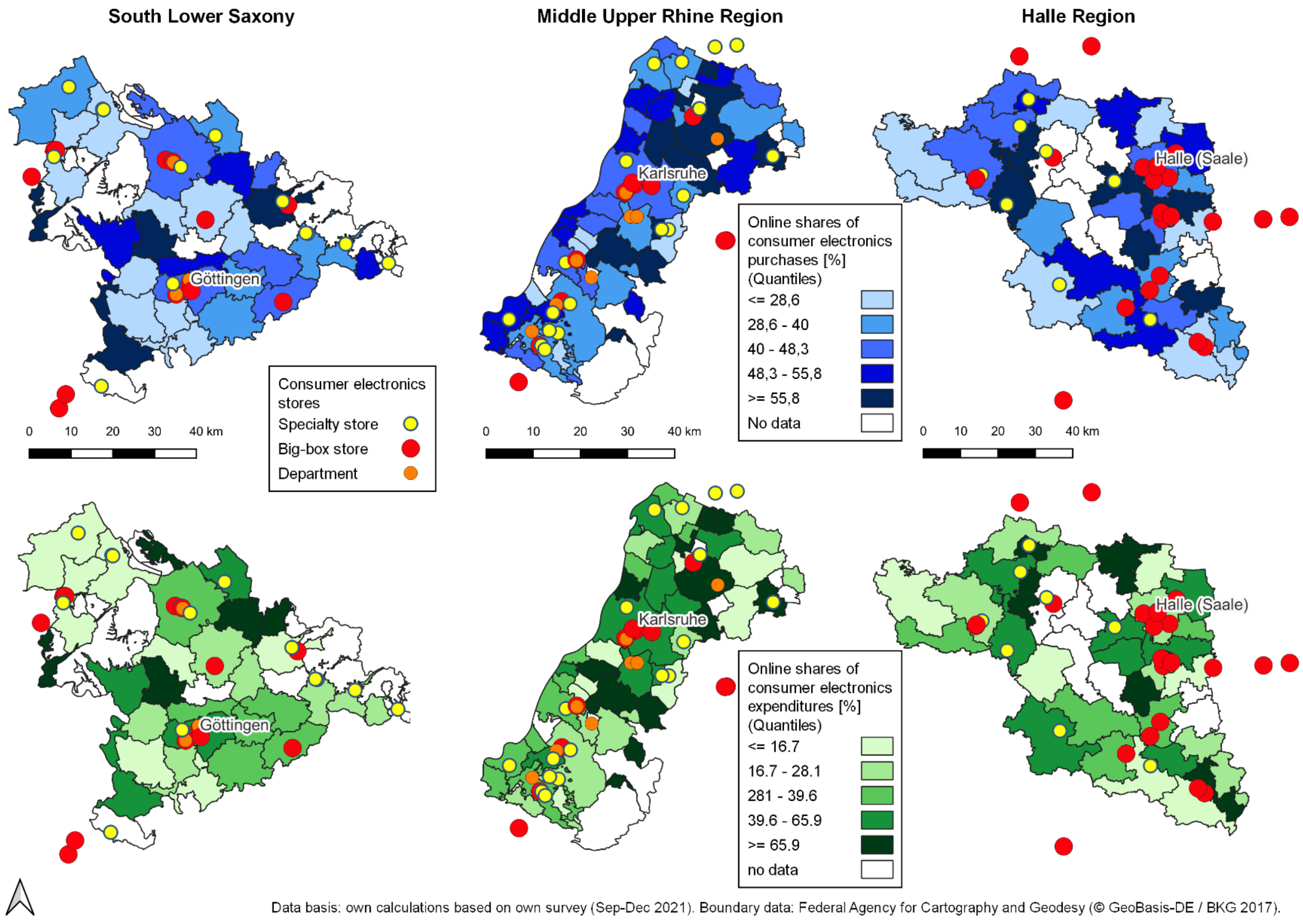

Figure S3: Consumer electronics purchases and expenditures separated by study area at the municipality level.

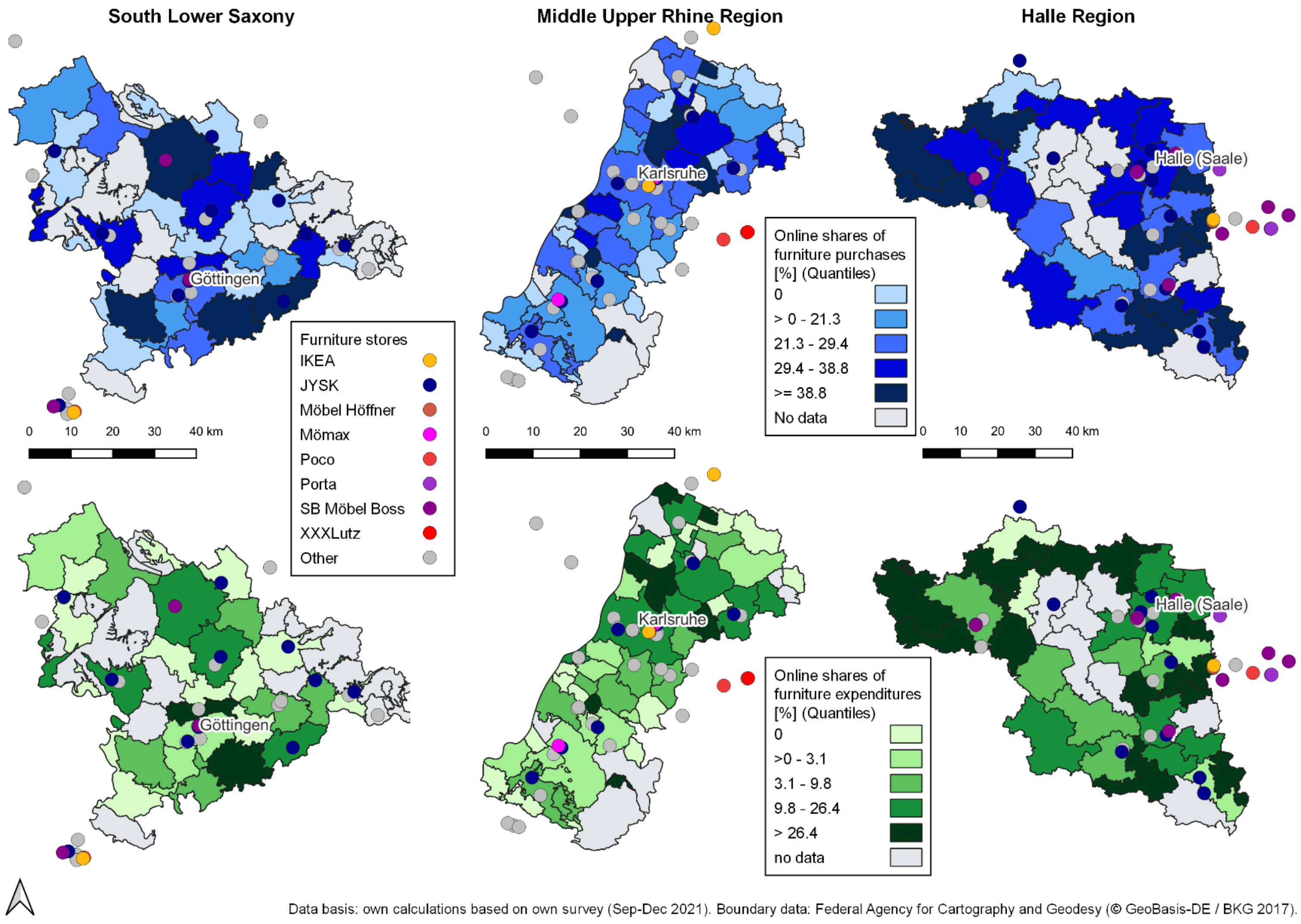

Figure S4: Furniture purchases and expenditures separated by study area at the municipality level.

Table S4: Relative frequencies and factor loadings of attitude items (non-missing only).

Item		Relative frequency [%]				Factor loadings	
		Agree	Rather agree	Rather disagree	Disagree	Pro online (LV 1)	Physical shopping pleasure (LV 2)
1	I often order products on the internet	25.40	21.96	25.77	26.87	–0.395	**0.678**
2	Online shopping is associated with risks	25.48	34.02	31.62	8.88	**0.677**	–0.001
3	Bank card/credit card fraud is one of the reasons why I don't like online shopping	20.20	17.57	30.76	31.47	**0.692**	–0.270
4	The internet has more cons than pros	10.35	19.15	40.28	30.18	**0.535**	–0.323
5	A disadvantage of online shopping is that I cannot physically examine the products	61.52	25.52	8.45	4.52	**0.631**	0.034
6	Online shopping facilitates the comparison of prices and products	50.57	34.41	9.13	5.90	0.052	**0.722**
7	The risk of receiving a wrong product is one of the main reasons why I don't like online shopping	15.80	18.30	37.35	28.55	**0.654**	–0.300
8	No matter if I buy online or in-store: Before buying, I get informed via internet about products and compare prices	36.19	28.52	18.37	16.92	–0.034	**0.717**
9	Online shopping affects the environment, e.g. by transportation	39.58	32.43	20.67	7.32	**0.590**	0.076
10	Online shopping facilitates poor working conditions, e.g. for the delivery employees	40.31	37.65	17.05	4.99	**0.657**	0.105
11	Shopping usually is an annoying duty	18.03	25.09	33.24	23.64	0.100	**0.450**
12	I like to visit shops, even if I don't want to buy something, just for looking around	24.04	20.70	25.73	29.53	0.207	**–0.317**
13	I feel to have no control of my data in the internet	25.33	28.42	29.33	16.92	**0.627**	–0.106
14	I feel that my personal data are sufficiently protected inside and outside the internet	8.12	27.45	43.36	21.07	**–0.310**	0.100
15	In general, the protection of my personal data is very important for me	63.51	27.11	7.03	2.34	**0.363**	–0.012
Cronbach's α						0.79	0.31
Cumulative % of explained variance						38.45	

Note: Values in bold indicate assignment of an item to a factor.

Table S5: Relative frequencies and factor loadings of the Perceived Vulnerability to Disease (PVD) scale (non-missing only).

		**Relative frequency [%]**				**Factor loadings**	
Item		Agree	Rather agree	Rather disagree	Disagree	Perceived Infectability (LV 1)	Germ Aversion (LV 2)
1	It really bothers me when people sneeze without covering their mouths *	74.28	17.13	6.20	2.39	0.008	**0.621**
2	If an illness is "going around", I will get it *	2.04	8.67	56.08	33.21	**0.657**	0.091
3	I am comfortable sharing a water bottle with a friend	23.30	23.78	24.35	28.57	–0.049	**–0.589**
4	My past experiences make me believe I am not likely to get sick even when my friends are sick	20.12	41.47	29.92	8.49	**–0.595**	–0.167
5	I have a history of susceptibility to infectious disease *	6.12	11.51	45.24	37.13	**0.789**	–0.020
6	I prefer to wash my hands pretty soon after shaking someone's hand *	23.17	25.67	34.26	16.91	0.161	**0.678**
7	In general, I am very susceptible to colds, flu and other infectious diseases *	5.38	10.40	43.00	41.21	**0.834**	0.023
8	I dislike wearing used clothes because you do not know what the last person who wore it was like *	33.89	18.43	23.27	24.41	0.025	**0.567**
9	I am more likely than the people around me to catch an infectious disease *	3.54	7.40	39.82	49.25	**0.761**	0.089
10	My hands do not feel dirty after touching money	23.23	25.70	29.87	21.21	–0.046	**–0.310**
11	I am unlikely to catch a cold, flu or other illness, even if it is "going around"	30.83	36.27	23.99	8.91	**–0.678**	–0.042
12	It does not make me anxious to be around sick people	25.10	37.35	27.90	9.65	**–0.384**	–0.280
13	My immune system protects me from most illnesses that other people get	22.23	52.66	19.06	6.01	**–0.698**	–0.064
Cronbach's α						0.83	0.47
Cumulative % of explained variance						42.68	

Note: Some items (*) were recoded (analogous to Duncan et al. 2009). Values in bold indicate assignment of an item to a factor.

Table S6: Consumer electronics stores – Modeling results for survey area 1 (South Lower Saxony).

**Explanatory variables**	**2019**		**2021**		
	**Participation equation**	**Expenditure equation**	**Participation equation A**	**Participation equation B**	**Expenditure equation**
**Store choice**					
Store attraction					
ln number of items$_j$	0.649*** (0.142)	0.082*** (0.009)	0.841*** (0.214)	0.833*** (0.214)	0.692*** (0.013)
Dummy MC with integrated online shop$_j$	1.279*** (0.480)	–0.640*** (0.026)	–0.173 (0.572)	–0.151 (0.572)	–0.553*** (0.029)
Dummy MC with BIPOS$_j$	–0.839** (0.411)	–0.148*** (0.021)	0.019 (0.376)	0.009 (0.377)	–0.120*** (0.024)
Dummy Saturn$_j$	–1.067** (0.501)	–0.285*** (0.031)	–0.410 (0.663)	–0.432 (0.663)	–0.242*** (0.037)
Dummy Media Markt$_j$	–1.463*** (0.562)	0.452*** (0.033)	0.157 (0.572)	0.143 (0.572)	–0.277*** (0.028)
Dummy Expert$_j$	–2.170*** (0.549)	0.506*** (0.032)	–0.582 (0.592)	–0.590 (0.591)	–0.143*** (0.028)
Dummy Euronics$_j$	–2.007*** (0.523)	–0.139*** (0.033)	–0.052 (0.480)	–0.048 (0.479)	0.724*** (0.023)
Dummy Electronic Partner$_j$	–1.700*** (0.655)	1.227*** (0.031)	0.168 (0.923)	0.139 (0.924)	2.566*** (0.053)
Dummy not full range$_j$	–1.729*** (0.341)	0.233*** (0.019)	–0.333 (0.480)	–0.351 (0.481)	0.147*** (0.029)
Dummy located in shopping mall$_j$	–0.262 (0.335)	0.348*** (0.017)	–1.253*** (0.411)	–1.244*** (0.412)	–0.153*** (0.022)
ln clustering$_j$+0.0001	0.058* (0.033)	–0.042*** (0.002)	0.062 (0.040)	0.061 (0.040)	0.035*** (0.002)
Dummy online store$_j$	–0.165 (2.003)	–0.536*** (0.108)	6.126** (2.479)	6.403** (2.491)	7.381*** (0.129)
ln number of items$_j$ x Dummy online store$_j$	–0.320* (0.176)	–0.022** (0.011)	–0.582*** (0.223)	–0.574** (0.224)	–0.747*** (0.013)
Shopping transaction costs					
Travel time$_{ij}$	–0.127*** (0.009)	0.008*** (0.001)	–0.063*** (0.006)	–0.063*** (0.006)	0.002*** (0.0001)
Delivery time$_j$	–0.723** (0.327)	–0.542*** (0.026)	–0.916*** (0.242)	–0.922*** (0.242)	–0.229*** (0.014)
Delivery charges$_j$	–0.145 (0.127)	0.156*** (0.008)	–0.234*** (0.057)	–0.235*** (0.057)	0.006** (0.003)
**Channel choice**					
Socio-demographic and spatial consumer attributes					
Dummy place of residence is large city$_i$	–0.923*** (0.238)	–	–0.461* (0.246)	–0.465* (0.247)	–
Dummy online store$_j$ x Dummy place of residence is large city$_i$	1.102*** (0.345)	–	0.038 (0.386)	0.005 (0.388)	–
Dummy age < 25$_i$	0.090 (0.295)	–	–0.435 (0.472)	–0.404 (0.474)	–
Dummy age >= 65$_i$	0.069 (0.292)	–	–0.140 (0.278)	–0.069 (0.282)	–

Dummy male$_i$	–0.168 (0.183)	–	0.043 (0.181)	0.037 (0.182)	–
Dummy employed$_i$	–0.102 (0.257)	–	–0.352 (0.260)	–0.381 (0.262)	–
Dummy online store$_j$ x Dummy age < 25$_i$	–0.108 (0.447)	–	0.686 (0.642)	0.607 (0.646)	–
Dummy online store$_j$ x Dummy age >= 65$_i$	–1.527*** (0.534)	–	–1.165** (0.496)	–1.221** (0.501)	–
Dummy online store$_j$ x Dummy male$_i$	0.273 (0.297)	–	0.028 (0.290)	0.048 (0.292)	–
Dummy online store$_j$ x Dummy employed$_i$	0.115 (0.395)	–	0.684* (0.406)	0.732* (0.409)	–
Shopping attitudes					
LV pro online$_i$	–0.112 (0.094)	–	–0.212** (0.096)	–0.223** (0.099)	–
Dummy online store$_j$ x LV pro online$_i$	0.512*** (0.158)	–	0.555*** (0.167)	0.534*** (0.171)	–
Attitudes towards pandemic situation					
LV perceived infectability$_i$	–	–	–	–0.040 (0.092)	–
LV germ aversion$_i$	–	–	–	–0.161 (0.108)	–
Face mask aversion$_i$	–	–	–	–0.007 (0.076)	–
Dummy online store$_j$ x LV perceived infectability$_i$	–	–	–	0.056 (0.149)	–
Dummy online store$_j$ x LV germ aversion$_i$	–	–	–	0.064 (0.167)	–
Dummy online store$_j$ x Face mask aversion$_i$	–	–	–	–0.168 (0.125)	–
ln expenditures$_i$	–	0.753*** (0.004)	–	–	1.060*** (0.002)
Constant	–4.523*** (1.085)	0.815*** (0.064)	–8.982*** (1.925)	–8.961*** (1.931)	–7.054*** (0.109)
Observations	11,484		7,596		
Log Likelihood	–20,560.10		–48,585.46 (A) / –48,582.42 (B)		
AIC	41,214.2		97,272.93 (A) / 97,278.84 (B)		

Note: * $p < 0.1$; ** $p < 0.05$; *** $p < 0.01$. Coefficient standard errors in parentheses. MC = Multi-channel retailer, LV = latent variable. Data for 2019 from Wieland (2021a).

Table S7: Consumer electronics stores – Modeling results for survey area 3 (Regional planning association Halle).

Explanatory variables	2021<br>Participation equation A	 Participation equation B	 Expenditure equation
**Store choice**			
Store attraction			
ln number of items$_j$	–0.362** (0.146)	–0.356** (0.147)	0.164*** (0.008)
Dummy MC with integrated online shop$_j$	–1.712*** (0.554)	–1.708*** (0.554)	–1.819*** (0.024)
Dummy MC with BIPOS$_j$	0.292 (0.522)	0.292 (0.522)	1.013*** (0.020)
Dummy Saturn$_j$	1.083*** (0.335)	1.074*** (0.336)	0.017 (0.017)
Dummy Media Markt$_j$	2.068*** (0.284)	2.065*** (0.284)	0.235*** (0.014)
Dummy Expert$_j$	0.158 (0.297)	0.149 (0.297)	0.047*** (0.014)
Dummy Euronics$_j$	-0.215 (0.223)	–0.216 (0.223)	0.301*** (0.008)
Dummy not full range$_j$	0.973* (0.578)	0.973* (0.578)	–0.496*** (0.046)
Dummy located in shopping mall$_j$	–0.566*** (0.193)	–0.570*** (0.193)	0.639*** (0.008)
ln clustering$_j$+0.0001	–0.080 (0.075)	–0.082 (0.075)	0.074*** (0.002)
Dummy online store$_j$	–8.721*** (1.894)	–8.405*** (1.904)	2.677*** (0.091)
ln number of items$_j$ x Dummy online store$_j$	0.625*** (0.151)	0.620*** (0.151)	–0.159*** (0.008)
Shopping transaction costs			
Travel time$_{ij}$	–0.083*** (0.005)	–0.084*** (0.005)	–0.003*** (0.0002)
Delivery time$_j$	–0.422*** (0.109)	–0.422*** (0.109)	0.022*** (0.003)
Delivery charges$_j$	–0.195*** (0.037)	–0.195*** (0.037)	–0.130*** (0.002)
**Channel choice**			
Socio-demographic and spatial consumer attributes			
Dummy place of residence is large city$_i$	0.136 (0.146)	0.135 (0.148)	–
Dummy online store$_j$ x Dummy place of residence is large city$_i$	–0.193 (0.212)	–0.205 (0.214)	–
Dummy age < 25$_i$	–0.699** (0.322)	–0.639** (0.325)	–
Dummy age >= 65$_i$	0.101 (0.223)	0.111 (0.231)	–
Dummy male$_i$	0.309** (0.134)	0.285** (0.136)	–

Dummy employed$_i$	0.227<br>(0.215)	0.214<br>(0.216)	–
Dummy online store$_j$ x Dummy age < 25$_i$	1.079***<br>(0.404)	0.982**<br>(0.410)	–
Dummy online store$_j$ x Dummy age >= 65$_i$	–0.779**<br>(0.331)	–0.750**<br>(0.342)	–
Dummy online store$_j$ x Dummy male$_i$	–0.398**<br>(0.199)	–0.371*<br>(0.201)	–
Dummy online store$_j$ x Dummy employed$_i$	–0.149<br>(0.295)	–0.123<br>(0.297)	–
Shopping attitudes			
LV pro online$_i$	–0.267***<br>(0.074)	–0.244***<br>(0.078)	–
Dummy online store$_j$ x LV pro online$_i$	0.595***<br>(0.110)	0.564***<br>(0.113)	–
Attitudes towards pandemic situation			
LV perceived infectability$_i$	–	–0.002<br>(0.065)	–
LV germ aversion$_i$	–	–0.018<br>(0.070)	–
Face mask aversion$_i$	–	0.103*<br>(0.055)	–
Dummy online store$_j$ x LV perceived infectability$_i$	–	0.012<br>(0.095)	–
Dummy online store$_j$ x LV germ aversion$_i$	–	–0.016<br>(0.104)	–
Dummy online store$_j$ x Face mask aversion$_i$	–	–0.144*<br>(0.082)	–
ln expenditures$_i$	–	–	1.036***<br>(0.001)
Constant	3.034**<br>(1.466)	2.779*<br>(1.474)	–2.740***<br>(0.079)
Observations	16,834		
Log Likelihood	–79,987.38 (A) / –79,985.24 (B)		
AIC	160072.80 (A) / 160080.5 (B)		

Note: * p < 0.1; ** p < 0.05; *** p < 0.01. Coefficient standard errors in parentheses. MC = Multi-channel retailer, LV = latent variable.

Table S8: Furniture stores – Modeling results for survey area 1 (South Lower Saxony).

Explanatory variables	2019		2021		
	**Participation equation**	**Expenditure equation**	**Participation equation A**	**Participation equation B**	**Expenditure equation**
**Store choice**					
Store attraction					
ln number of items$_j$	0.918*** (0.172)	–0.361*** (0.003)	0.560** (0.221)	0.576** (0.224)	–0.124*** (0.005)
Dummy Cross-Channel Retailer$_j$	0.857* (0.520)	0.215*** (0.008)	0.322 (0.659)	0.359 (0.661)	–0.176*** (0.014)
Dummy JYSK$_j$	–0.195 (0.591)	–2.325*** (0.016)	–0.132 (0.763)	-0.133 (0.767)	–0.211*** (0.022)
Dummy IKEA$_j$	2.034*** (0.593)	–0.061*** (0.013)	2.561*** (0.768)	2.652*** (0.772)	0.309*** (0.018)
Dummy Poco$_j$	–1.410 (0.921)	0.041 (0.048)	–0.284 (1.024)	–0.227 (1.026)	–1.002*** (0.078)
Dummy SB Möbel Boss$_j$	–1.121* (0.673)	–0.849*** (0.025)	–0.613 (0.823)	–0.626 (0.826)	0.487*** (0.023)
Dummy Sconto$_j$	–1.044 (0.737)	–0.660*** (0.036)	–0.563 (0.902)	–0.586 (0.905)	–0.558*** (0.030)
Dummy XXXLutz$_j$	–0.965 (1.029)	0.791*** (0.058)	–	–	–
Dummy Amazon$_j$	0.103 (1.460)	–3.910*** (0.165)	–1.459 (0.961)	–1.492 (0.961)	–3.442*** (0.068)
Dummy eBay$_j$	0.353 (1.416)	–2.794*** (0.161)	–2.416** (1.126)	–2.456** (1.126)	–1.447*** (0.075)
ln clustering$_j$+0.0001	–0.012 (0.030)	–0.048*** (0.001)	–0.091** (0.044)	–0.098** (0.045)	–0.050*** (0.001)
Dummy online store$_j$	2.258 (2.839)	–8.555*** (0.263)	–3.199 (3.127)	–3.237 (3.171)	–4.928*** (0.150)
ln number of items$_j$ x Dummy online store$_j$	–0.772*** (0.264)	0.761*** (0.025)	–0.218 (0.265)	–0.228 (0.268)	0.300*** (0.012)
Shopping transaction costs					
Travel time$_{ij}$	–0.088*** (0.007)	–0.002*** (0.0002)	–0.059*** (0.007)	–0.062*** (0.008)	–0.0002 (0.0003)
Delivery charges$_j$	–0.0002 (0.023)	–0.027*** (0.001)	0.009 (0.015)	0.009 (0.015)	0.010*** (0.001)
Dummy delivery charges based on order value$_j$	–2.527** (1.156)	2.421*** (0.091)	–3.219*** (0.868)	–3.349*** (0.874)	–0.341*** (0.042)
Dummy free delivery from a certain order value$_j$	–1.090 (1.483)	3.030*** (0.188)	1.230 (1.153)	1.320 (1.155)	0.494*** (0.088)
**Channel choice**					
Socio-demographic and spatial consumer attributes					
Dummy place of residence is large city$_i$	–0.600*** (0.227)	–	–0.013 (0.298)	–0.182 (0.312)	–
Dummy online store$_j$ x Dummy place of residence is large city$_i$	1.203*** (0.440)	–	–0.342 (0.585)	–0.259 (0.605)	–
Dummy age < 25$_i$	0.222 (0.315)	–	0.752 (0.486)	0.947* (0.506)	–

Dummy age >= 65$_i$	–0.199<br>(0.318)	–	–0.398<br>(0.419)	–0.269<br>(0.417)	–
Dummy male$_i$	–0.156<br>(0.194)	–	0.336<br>(0.245)	0.277<br>(0.247)	–
Dummy employed$_i$	0.151<br>(0.263)	–	–0.345<br>(0.357)	–0.554<br>(0.369)	–
Dummy online store$_j$ x Dummy age < 25$_i$	–1.381*<br>(0.763)	–	–1.037<br>(1.176)	–1.353<br>(1.191)	–
Dummy online store$_j$ x Dummy age >= 65$_i$	–0.994<br>(0.750)	–	0.977<br>(0.920)	0.921<br>(0.913)	–
Dummy online store$_j$ x Dummy male$_i$	–0.062<br>(0.431)	–	–0.641<br>(0.455)	–0.611<br>(0.458)	–
Dummy online store$_j$ x Dummy employed$_i$	–0.347<br>(0.538)	–	1.619*<br>(0.839)	1.805**<br>(0.847)	–
Shopping attitudes					
LV pro online$_i$	–0.043<br>(0.105)	–	–0.093<br>(0.146)	–0.124<br>(0.149)	–
Dummy online store$_j$ x LV pro online$_i$	0.727***<br>(0.238)	–	0.385<br>(0.263)	0.420<br>(0.272)	–
Attitudes towards pandemic situation					
LV perceived infectability$_i$	–	–	–	0.213<br>(0.136)	–
LV germ aversion$_i$	–	–	–	–0.484***<br>(0.150)	–
Face mask aversion$_i$	–	–	–	–0.112<br>(0.108)	–
Dummy online store$_j$ x LV perceived infectability$_i$	–	–	–	0.013<br>(0.240)	–
Dummy online store$_j$ x LV germ aversion$_i$	–	–	–	0.483*<br>(0.250)	–
Dummy online store$_j$ x Face mask aversion$_i$	–	–	–	–0.062<br>(0.200)	–
ln expenditures$_i$	–	0.829***<br>(0.002)	--	–	1.075***<br>(0.001)
Constant	–7.988***<br>(1.546)	4.334***<br>(0.036)	–5.766***<br>(2.109)	–5.484**<br>(2.149)	0.444***<br>(0.042)
Observations	5,771		3,744		
Log Likelihood	–50,693.77		–21,756.47 (A) / –21,749.40 (B)		
AIC	101,485.50		43,606.94 (A) / 43,604.79 (B)		

Note: * p < 0.1; ** p < 0.05; *** p < 0.01. Coefficient standard errors in parentheses. LV = latent variable. Data for 2019 from Wieland (2023).

Table S9: Furniture stores – Modeling results for survey area 3 (Regional planning association Halle).

Explanatory variables	2021 Participation equation A	2021 Participation equation B	2021 Expenditure equation
**Store choice**			
Store attraction			
ln number of items$_j$	0.514*** (0.077)	0.514*** (0.077)	0.244*** (0.002)
Dummy Cross-Channel retailer$_j$	0.187 (0.277)	0.186 (0.277)	–0.716*** (0.010)
Dummy JYSK$_j$	–0.078 (0.409)	-0.079 (0.409)	1.141*** (0.013)
Dummy IKEA$_j$	2.515*** (0.257)	2.513*** (0.257)	0.157*** (0.009)
Dummy Mömax$_j$	0.459 (0.429)	0.459 (0.429)	0.765*** (0.016)
Dummy Roller$_j$	–0.212 (0.329)	–0.211 (0.329)	0.617*** (0.011)
Dummy Amazon$_j$	–1.095** (0.521)	–1.093** (0.521)	0.623*** (0.028)
Dummy eBay$_j$	–1.443*** (0.548)	–1.442*** (0.548)	–0.396*** (0.034)
ln clustering$_j$+0.0001	–0.023 (0.048)	–0.023 (0.048)	–0.060*** (0.001)
Dummy online store$_j$	0.095 (1.730)	–0.204 (1.746)	–1.010*** (0.079)
ln number of items$_j$ x Dummy online store$_j$	–0.147 (0.118)	–0.147 (0.118)	–0.149*** (0.006)
Shopping transaction costs			
Travel time$_{ij}$	–0.035*** (0.005)	–0.034*** (0.005)	–0.002*** (0.0001)
Delivery charges$_j$	0.008 (0.012)	0.008 (0.012)	0.029*** (0.001)
Dummy delivery charges based on order value$_j$	–2.530*** (0.439)	–2.529*** (0.439)	0.079*** (0.024)
Dummy free delivery from a certain order value$_j$	0.219 (0.620)	0.218 (0.620)	–0.227*** (0.034)
**Channel choice**			
Socio-demographic and spatial consumer attributes			
Dummy place of residence is large city$_i$	–0.104 (0.168)	–0.128 (0.170)	–
Dummy online store$_j$ x Dummy place of residence is large city$_i$	0.085 (0.272)	0.141 (0.278)	–
Dummy age < 25$_i$	0.158 (0.304)	0.140 (0.306)	–
Dummy age >= 65$_i$	0.157 (0.282)	0.191 (0.293)	–
Dummy male$_i$	0.085 (0.162)	0.096 (0.164)	–

Dummy employed$_i$	0.368<br>(0.252)	0.396<br>(0.256)	–
Dummy online store$_j$ x Dummy age < 25$_i$	0.201<br>(0.443)	0.232<br>(0.449)	–
Dummy online store$_j$ x Dummy age >= 65$_i$	–0.886**<br>(0.433)	–0.902**<br>(0.451)	–
Dummy online store$_j$ x Dummy male$_i$	–0.121<br>(0.263)	–0.154<br>(0.265)	–
Dummy online store$_j$ x Dummy employed$_i$	–0.746**<br>(0.361)	–0.788**<br>(0.365)	–
Shopping attitudes			
LV pro online$_i$	–0.129<br>(0.089)	–0.136<br>(0.091)	–
Dummy online store$_j$ x LV pro online$_i$	0.327**<br>(0.145)	0.326**<br>(0.147)	–
Attitudes towards pandemic situation			
LV perceived infectability$_i$	–	–0.002<br>(0.079)	–
LV germ aversion$_i$	–	–0.014<br>(0.083)	–
Face mask aversion$_i$	–	–0.056<br>(0.067)	–
Dummy online store$_j$ x LV perceived infectability$_i$	–	–0.039<br>(0.127)	–
Dummy online store$_j$ x LV germ aversion$_i$	–	–0.041<br>(0.132)	–
Dummy online store$_j$ x Face mask aversion$_i$	–	0.140<br>(0.108)	–
ln expenditures$_i$	–	–	1.049***<br>(0.001)
Constant	–7.683***<br>(0.884)	–7.580***<br>(0.894)	–2.408***<br>(0.025)
Observations	10,608		
Log Likelihood	–74,003.510 (A) / –74,002.280 (B)		
AIC	148,097.0 (A) / 148,106.6 (B)		

Note: * $p < 0.1$; ** $p < 0.05$; *** $p < 0.01$. Coefficient standard errors in parentheses. LV = latent variable.

References

Duncan LA, Schaller M, Park JH (2009) Perceived vulnerability to disease: Development and validation of a 15-item self-report instrument. *Pers. Individ. Differ.* 47(6), 541-546. https://doi.org/10.1016/j.paid.2009.05.001.

Wieland T (2021) Identifying the Determinants of Store Choice in a Multi-Channel Environment: A Hurdle Model Approach. *Pap. Appl. Geogr.* 7(4): 343-371. https://doi.org/10.1080/23754931.2021.1895875.

Wieland T (2023) A Micro-Econometric Store Choice Model Incorporating Multi- and Omni-Channel Shopping: The Case of Furniture Retailing in Germany. *Geogr. Anal.* 55 (1):3-30. https://doi.org/10.1111/gean.12308.
